# Supplementary material for: Key Members of the CmPn as Biomarkers Distinguish Histological and Immune Subtypes of Hepatic Cancers
Source: Diagnostics (Basel). 2023 Mar 7;13(6):1012. doi: 10.3390/diagnostics13061012 (PMC10047786; doi:10.3390/diagnostics13061012)
Supplement: Supplementary file 1 [file diagnostics-13-01012-s001.zip › diagnostics-2213936-Suppl figures.pdf]

Differential expression of key CmPn members distinguishes histological and immune subtypes of hepatic cancers

Johnathan Abou-Fadel, Victoria Reid, Alexander Le, Jun Zhang *

Department of Molecular and Translational Medicine (MTM)

Texas Tech University Health Science Center El Paso, El Paso, TX 79905 USA

*Correspondence: jun.zhang2000@gmail.com; Tel.: +1-(915)-215-4197

Legends

Suppl. Figures

Suppl. Figure S1. Differentially expressed genes across immune subtypes for Hepatocellular Carcinoma (HCC). A) *Comparisons between wound healing (C1) and IFN-γ dominant (C2) subtypes.* Sample size is shown for each comparing group (top left panel) along with a scatter plot (middle left panel) of differentially expressed genes (DEGs). Results of the gene ontology enrichment analysis comparing C1 and C2 are provided for biological processes (lower left panel), molecular functions (upper right panel), reactome pathways (middle right panel), and KEGG pathways (lower right panel). **B)** *Comparisons between wound healing (C1) and inflammatory (C3) subtypes*. Sample size is shown for each comparing group (top left panel) along with a scatter plot (middle left panel) of DEGs. Results of the gene ontology enrichment analysis comparing C1 and C3 are provided for biological processes (lower left panel), molecular functions (upper right panel), reactome pathways (middle right panel), and KEGG pathways (lower right panel). **C)** *Comparisons between wound healing (C1) and lymphocyte depleted (C4) subtypes*. Sample size is shown for each comparing group (top left panel) along with a scatter plot (middle left panel) of DEGs. Results of the gene ontology enrichment analysis comparing C1 and C4 are provided for biological processes (lower left panel), molecular functions (upper right panel), reactome pathways (middle right panel), and KEGG pathways (lower right panel). **D)** *Comparisons between IFN-γ dominant (C2) and inflammatory (C3) subtypes.* Sample size is shown for each comparing group (top left panel) along with a scatter plot (middle left panel) of DEGs. Results of the gene ontology enrichment analysis comparing C2 and C3 are provided for biological processes (lower left panel), molecular functions (upper right panel), reactome pathways (middle right panel), and KEGG pathways (lower right panel). **E)** *Comparisons between IFN-γ dominant (C2) and lymphocyte depleted (C4) subtypes.* Sample size is shown for each comparing group (top left panel) along with a scatter plot (middle left panel) of DEGs. Results of the gene ontology enrichment analysis comparing C2 and C4 are provided for biological processes (lower left panel), molecular functions

(upper right panel), reactome pathways (middle right panel), and KEGG pathways (lower right panel). **F)** *Comparisons between inflammatory (C3) and lymphocyte depleted (C4) subtypes.* Sample size is shown for each comparing group (top left panel) along with a scatter plot (middle left panel) of DEGs. Results of the gene ontology enrichment analysis comparing C3 and C4 are provided for biological processes (lower left panel), molecular functions (upper right panel), reactome pathways (middle right panel), and KEGG pathways (lower right panel). For all panels, scatter plots display the log2-fold changes and statistical significance of each gene calculated by performing a differential gene expression analysis. Genes in volcano plots with logFC>1.5 and p-value<0.05 are in red while genes with logFC<-1.5 and p-value<0.05 are in blue. All enrichment analysis results were generated using Enrichr where the x axis indicates the -log10(P-value); the top 15 pathways are provided and color-coded (red-up/blue-down) and pathways with p-values<0.05 and FDR<0.1 are bolded.

Suppl. Figure S2. Differentially expressed genes across immune subtypes for Cholangiocarcinoma (CCA). A) *Comparisons between wound healing (C1) and IFN-γ dominant (C2) subtypes.* Sample size is shown for each comparing group (top left panel) along with a scatter plot (middle left panel) of differentially expressed genes (DEGs). Results of the gene ontology enrichment analysis comparing C1 and C2 are provided for biological processes (lower left panel), molecular functions (upper right panel), reactome pathways (middle right panel), and KEGG pathways (lower right panel). **B)** *Comparisons between wound healing (C1) and inflammatory (C3) subtypes*. Sample size is shown for each comparing group (top left panel) along with a scatter plot (middle left panel) of DEGs. Results of the gene ontology enrichment analysis comparing C1 and C3 are provided for biological processes (lower left panel), molecular functions (upper right panel), reactome pathways (middle right panel), and KEGG pathways (lower right panel). **C)** *Comparisons between wound healing (C1) and lymphocyte depleted (C4) subtypes*. Sample size is shown for each comparing group (top left panel) along with a scatter plot (middle left panel) of DEGs. Results of

the gene ontology enrichment analysis comparing C1 and C4 are provided for biological processes (lower left panel), molecular functions (upper right panel), reactome pathways (middle right panel), and KEGG pathways (lower right panel). **D)** *Comparisons between IFN-γ dominant (C2) and inflammatory (C3) subtypes.* Sample size is shown for each comparing group (top left panel) along with a scatter plot (middle left panel) of DEGs. Results of the gene ontology enrichment analysis comparing C2 and C3 are provided for biological processes (lower left panel), molecular functions (upper right panel), reactome pathways (middle right panel), and KEGG pathways (lower right panel). **E)** *Comparisons between IFN-γ dominant (C2) and lymphocyte depleted (C4) subtypes.* Sample size is shown for each comparing group (top left panel) along with a scatter plot (middle left panel) of DEGs. Results of the gene ontology enrichment analysis comparing C2 and C4 are provided for biological processes (lower left panel), molecular functions (upper right panel), reactome pathways (middle right panel), and KEGG pathways (lower right panel). **F)** *Comparisons between inflammatory (C3) and lymphocyte depleted (C4) subtypes.* Sample size is shown for each comparing group (top left panel) along with a scatter plot (middle left panel) of DEGs. Results of the gene ontology enrichment analysis comparing C3 and C4 are provided for biological processes (lower left panel), molecular functions (upper right panel), reactome pathways (middle right panel), and KEGG pathways (lower right panel). For all panels, scatter plots display the log2-fold changes and statistical significance of each gene calculated by performing a differential gene expression analysis. Genes in volcano plots with logFC>1.5 and p-value<0.05 are in red while genes with logFC<-1.5 and p-value<0.05 are in blue. All enrichment analysis results were generated using Enrichr where the x axis indicates the -log10(P-value); the top 15 pathways are provided and color-coded (red-up/blue-down) and pathways with p-values<0.05 and FDR<0.1 are bolded.

Suppl. Figure S3. RNAseq expression profiling for key CmPn players integrating sociological, follow-up, and clinical survival data for hepatic cancers: We investigated key CSC, mPRs and nPRs (CmPn) players expression analysis (along with established liver cancer biomarker *AFP*) using demographic and follow-up

data to assess differential expression across major races and evaluate impact on tumor recurrence following treatment. **A)** Differential expression for key CmPn members, along with *AFP,* among the three most prevalent races diagnosed with CCAs (n=45). **B)** Differential expression for key CmPn members, along with *AFP,* among the five major locations for tumor recurrence in patients diagnosed with CCAs (n=20). **C)** Differential expression for key CmPn members, along with *AFP,* based on vital status for patients diagnosed with CCAs (n=45). **D1-2)** Differential expression of key CmPn members, along with *AFP,* for patients diagnosed with HCC (**D-1,** n=412) or patients diagnosed with CCAs (**D-2,** n=45). **E1-2)** Differential expression for key CmPn members, along with *AFP,* for residual tumor classification in patients diagnosed with HCCs (**E-1,** n=380) or for patients diagnosed with CCAs (**E-2,** n=41). For all graphs, X axis details genes profiled, while Y axis details Log2 batch effects normalized RNAseq expression data. All graphs were produced using the Xena platform.

Suppl. Figure S4. Hematoxylin and Eosin (H&E) imaging of microarray slides for normal and seven subtypes of hepatic cancers. H&E pre-stained microarray slides were purchased to visualize tissue patterns and subcellular structural differences between lesion subtypes. A representative normal healthy liver tissue (NORM) is displayed (Panel A) along with a representative hepatocellular carcinoma (HCC) tissue (panel B), cholangiocarcinoma (CCA) tissue (panel C), combined hepatocholangiocarcinoma (cHCC-CCA) tissue (panel D), adenosquamous carcinoma (ASC) tissue (panel E), undifferentiated pleomorphic sarcoma (UPS) tissue (panel F), clear cell carcinoma (CCC) tissue (panel G) and hepatic angiosarcoma (HAS) tissue (panel H). Tissues were imaged using a Nikon EclipseTi microscope with Images acquired using a 20X objective lens to ensure coverage of tissue.

Suppl. Figure S5. Immunofluorescence imaging of AFP and nPRs for hepatic cancers. Comparative protein expression patterns for AFP and nPRs were measured with immunofluorescence-labeled antibodies,

normalized against nuclear staining (DAPI), and quantified using Nikon Elements Analysis software. **A)** Representative IF images of AFP protein expression between NORM and CCA tissues is displayed, along with **n**ormalized quantification for all images. **B)** Normalized quantification for all images of nPRs protein expression among different liver cancer types and NORM tissue (images not shown).

Suppl. Tables

Suppl. Table S1. Databases used for NCI-GDC clinical profiling of hepatic cancers in Figure 1. Databases used by the Genomic Data Commons (GDC) data portal from the National Cancer Institute (NCI), are detailed in tab 1 for the general analysis of hepatic cancers without any gene filters (general clinical observations) or tab 2 for the filtered analysis to only analyze patient samples with differential expression for any of the key CmPn players (CmPn network associated clinical observations). The database of Genotypes and Phenotypes (dbGaP) study accession number is provided for each database.

Suppl. Table S2A. Raw RNAseq/enrichment results for normal healthy patients compared to HCC patients. The raw RNAseq data used to generate the differential expression analysis (volcano plot and corresponding table with CmPn expression differences) in Figure 2C-1 is provided in tab 1 with gene symbol in column A, log fold changes (logFC) in column B, average expression in column C, t-values in column D, P-value in column E and adjusted P-values in column F. The raw data for the corresponding Gene Ontology enrichment analysis performed using Enrichr is provided in tab 2 (Biological processes) and tab 3 (Molecular functions). The raw data for the corresponding KEGG pathways enrichment analysis performed using Enrichr is provided in tab 4. For Tabs 2-4, the terms, p-value, FDR, Z-score, combined score and corresponding genes are provided in columns A-F (up-regulated), respectively, or columns I-N (down-regulated), respectively. The top 15 terms (which are illustrated in the corresponding figure) are

color-coded (red for up regulated or blue for down regulated) and terms that had p-values <0.05 and FDR<0.1 are bolded.

Suppl. Table S2B. Raw RNAseq/enrichment results for normal healthy patients compared to CCA patients. The raw RNAseq data used to generate the differential expression analysis (volcano plot and corresponding table with CmPn expression differences) in Figure 2D-1 is provided in tab 1 with gene symbol in column A, log fold changes (logFC) in column B, average expression in column C, t-values in column D, P-value in column E and adjusted P-values in column F. The raw data for the corresponding Gene Ontology enrichment analysis performed using Enrichr is provided in tab 2 (Biological processes) and tab 3 (Molecular functions). The raw data for the corresponding KEGG pathways enrichment analysis performed using Enrichr is provided in tab 4. For Tabs 2-4, the terms, p-value, FDR, Z-score, combined score and corresponding genes are provided in columns A-F (up-regulated), respectively, or columns I-N (down-regulated), respectively. The top 15 terms (which are illustrated in the corresponding figure) are color-coded (red for up regulated or blue for down regulated) and terms that had p-values <0.05 and FDR<0.1 are bolded.

Suppl. Table S2C. Raw RNAseq/enrichment results for HCC compared to CCA patients. The raw RNAseq data used to generate the differential expression analysis (volcano plot and corresponding table with CmPn expression differences) in Figure 2E-1 is provided in tab 1 with gene symbol in column A, log fold changes (logFC) in column B, average expression in column C, t-values in column D, P-value in column E and adjusted P-values in column F. The raw data for the corresponding Gene Ontology enrichment analysis performed using Enrichr is provided in tab 2 (Biological processes) and tab 3 (Molecular functions). The raw data for the corresponding KEGG pathways enrichment analysis performed using Enrichr is provided in tab 4. For Tabs 2-4, the terms, p-value, FDR, Z-score, combined score and corresponding genes are

provided in columns A-F (up-regulated), respectively, or columns I-N (down-regulated), respectively. The top 15 terms (which are illustrated in the corresponding figure) are color-coded (red for up regulated or blue for down regulated) and terms that had p-values <0.05 and FDR<0.1 are bolded.

Suppl. Table S3. Antibodies and Microarray slides used for Immunofluorescence / H&E analysis. Details regarding application, antigen, gene, clone, manufacturer, catalog number, and primary and secondary dilutions (if applicable) for primary and secondary antibodies used are listed in tab 1. Information about microarray slides purchased for imaging are detailed in tab 2 with catalog #, details, manufacturer, and application. Abbreviations: IF, immunofluorescence; IHC, immunohistochemistry.

Suppl. Table S4A. Raw RNAseq/enrichment results for HCC Immune subtypes C1 v C2. The raw RNAseq data used to generate the differential expression analysis (volcano plot and corresponding table with CmPn expression differences) in Suppl. Fig 1A is provided in tab 1; gene symbol is in column A, log fold changes (logFC) in column B, average expression in column C, t-values in column D, P-values in column E and adjusted P-values in column F. The raw data for the corresponding Gene Ontology enrichment analysis performed using Enrichr is provided in tab 2 (Biological processes) and tab 3 (Molecular functions). The raw data for the corresponding signaling pathways enrichment analysis performed using Enrichr is provided in tab 4 (reactome signaling pathways) and tab 5 (KEGG signaling pathways). For tabs 2-5, the terms, p-value, FDR, Z-score, combined score and corresponding genes are provided in columns A-F (up-regulated), respectively, or columns I-N (down-regulated), respectively. The top 15 terms (which are illustrated in the corresponding figure) are color-coded (red for up regulated or blue for down regulated) and terms that had p-values <0.05 and FDR<0.1 are bolded.

Suppl. Table S4B. Raw RNAseq/enrichment results for HCC Immune subtypes C1 v C3. The raw RNAseq data used to generate the differential expression analysis (volcano plot and corresponding table with CmPn expression differences) in Suppl. Fig 1B is provided in tab 1; gene symbol is located in column A, log fold changes (logFC) in column B, average expression in column C, t-values in column D, P-value in column E and adjusted P-values in column F. The raw data for the corresponding Gene Ontology enrichment analysis performed using Enrichr is provided in tab 2 (Biological processes) and tab 3 (Molecular functions). The raw data for the corresponding signaling pathways enrichment analysis performed using Enrichr is provided in tab 4 (reactome signaling pathways) and tab 5 (KEGG signaling pathways). For tabs 2-5, the terms, p-value, FDR, Z-score, combined score and corresponding genes are provided in columns A-F (up regulated), respectively, or columns I-N (down regulated), respectively. The top 15 terms (which are illustrated in the corresponding figure) are color-coded (red for up regulated or blue for down regulated) and terms that had p-values <0.05 and FDR<0.1 are bolded.

Suppl. Table S4C. Raw RNAseq/enrichment results for HCC Immune subtypes C1 v C4. The raw RNAseq data used to generate the differential expression analysis (volcano plot and corresponding table with CmPn expression differences) in Suppl. Fig 1C is provided in tab 1; gene symbol is located in column A, log fold changes (logFC) in column B, average expression in column C, t-values in column D, P-value in column E and adjusted P-values in column F. The raw data for the corresponding Gene Ontology enrichment analysis performed using Enrichr is provided in tab 2 (Biological processes) and tab 3 (Molecular functions). The raw data for the corresponding signaling pathways enrichment analysis performed using Enrichr is provided in tab 4 (reactome signaling pathways) and tab 5 (KEGG signaling pathways). For tabs 2-5, the terms, p-value, FDR, Z-score, combined score and corresponding genes are provided in columns A-F (up regulated), respectively, or columns I-N (down regulated), respectively. The top 15 terms (which

are illustrated in the corresponding figure) are color-coded (red for up regulated or blue for down regulated) and terms that had p-values <0.05 and FDR<0.1 are bolded.

Suppl. Table S4D. Raw RNAseq/enrichment results for HCC Immune subtypes C2 v C3. The raw RNAseq data used to generate the differential expression analysis (volcano plot and corresponding table with CmPn expression differences) in Suppl. Fig 1D is provided in tab 1; gene symbol is located in column A, log fold changes (logFC) in column B, average expression in column C, t-values in column D, P-value in column E and adjusted P-values in column F. The raw data for the corresponding Gene Ontology enrichment analysis performed using Enrichr is provided in tab 2 (Biological processes) and tab 3 (Molecular functions). The raw data for the corresponding signaling pathways enrichment analysis performed using Enrichr is provided in tab 4 (reactome signaling pathways) and tab 5 (KEGG signaling pathways). For tabs 2-5, the terms, p-value, FDR, Z-score, combined score and corresponding genes are provided in columns A-F (up regulated), respectively, or columns I-N (down regulated), respectively. The top 15 terms (which are illustrated in the corresponding figure) are color-coded (red for up regulated or blue for down regulated) and terms that had p-values <0.05 and FDR<0.1 are bolded.

Suppl. Table S4E. Raw RNAseq/enrichment results for HCC Immune subtypes C2 v C4. The raw RNAseq data used to generate the differential expression analysis (volcano plot and corresponding table with CmPn expression differences) in Suppl. Fig 1E is provided in tab 1; gene symbol is located in column A, log fold changes (logFC) in column B, average expression in column C, t-values in column D, P-value in column E and adjusted P-values in column F. The raw data for the corresponding Gene Ontology enrichment analysis performed using Enrichr is provided in tab 2 (Biological processes) and tab 3 (Molecular functions). The raw data for the corresponding signaling pathways enrichment analysis performed using Enrichr is provided in tab 4 (reactome signaling pathways) and tab 5 (KEGG signaling pathways). For tabs

2-5, the terms, p-value, FDR, Z-score, combined score and corresponding genes are provided in columns A-F (up regulated), respectively, or columns I-N (down regulated), respectively. The top 15 terms (which are illustrated in the corresponding figure) are color-coded (red for up regulated or blue for down regulated) and terms that had p-values <0.05 and FDR<0.1 are bolded.

Suppl. Table S4F. Raw RNAseq/enrichment results for HCC Immune subtypes C3 v C4. The raw RNAseq data used to generate the differential expression analysis (volcano plot and corresponding table with CmPn expression differences) in Suppl. Fig 1F is provided in tab 1; gene symbol is located in column A, log fold changes (logFC) in column B, average expression in column C, t-values in column D, P-value in column E and adjusted P-values in column F. The raw data for the corresponding Gene Ontology enrichment analysis performed using Enrichr is provided in tab 2 (Biological processes) and tab 3 (Molecular functions). The raw data for the corresponding signaling pathways enrichment analysis performed using Enrichr is provided in tab 4 (reactome signaling pathways) and tab 5 (KEGG signaling pathways). For tabs 2-5, the terms, p-value, FDR, Z-score, combined score and corresponding genes are provided in columns A-F (up regulated), respectively, or columns I-N (down regulated), respectively. The top 15 terms (which are illustrated in the corresponding figure) are color-coded (red for up regulated or blue for down regulated) and terms that had p-values <0.05 and FDR<0.1 are bolded.

Suppl. Table S5A. Raw RNAseq/enrichment results for CCA Immune subtypes C1 v C2. The raw RNAseq data used to generate the differential expression analysis (volcano plot and corresponding table with CmPn expression differences) in Suppl. Fig 2A is provided in tab 1; gene symbol is located in column A, log fold changes (logFC) in column B, average expression in column C, t-values in column D, P-values in column E and adjusted P-values in column F. The raw data for the corresponding Gene Ontology enrichment analysis performed using Enrichr is provided in tab 2 (Biological processes) and tab 3 (Molecular

functions). The raw data for the corresponding signaling pathways enrichment analysis performed using Enrichr is provided in tab 4 (reactome signaling pathways) and tab 5 (KEGG signaling pathways). For tabs 2-5, the terms, p-value, FDR, Z-score, combined score and corresponding genes are provided in columns A-F (up-regulated), respectively, or columns I-N (down-regulated), respectively. The top 15 terms (which are illustrated in the corresponding figure) are color-coded (red for up regulated or blue for down regulated) and terms that had p-values <0.05 and FDR<0.1 are bolded.

Suppl. Table S5B. Raw RNAseq/enrichment results for CCA Immune subtypes C1 v C3. The raw RNAseq data used to generate the differential expression analysis (volcano plot and corresponding table with CmPn expression differences) in Suppl. Fig 2B is provided in tab 1; gene symbol is located in column A, log fold changes (logFC) in column B, average expression in column C, t-values in column D, P-value in column E and adjusted P-values in column F. The raw data for the corresponding Gene Ontology enrichment analysis performed using Enrichr is provided in tab 2 (Biological processes) and tab 3 (Molecular functions). The raw data for the corresponding signaling pathways enrichment analysis performed using Enrichr is provided in tab 4 (reactome signaling pathways) and tab 5 (KEGG signaling pathways). For tabs 2-5, the terms, p-value, FDR, Z-score, combined score and corresponding genes are provided in columns A-F (up regulated), respectively, or columns I-N (down regulated), respectively. The top 15 terms (which are illustrated in the corresponding figure) are color-coded (red for up regulated or blue for down regulated) and terms that had p-values <0.05 and FDR<0.1 are bolded.

Suppl. Table S5C. Raw RNAseq/enrichment results for CCA Immune subtypes C1 v C4. The raw RNAseq data used to generate the differential expression analysis (volcano plot and corresponding table with CmPn expression differences) in Suppl. Fig 2C is provided in tab 1; gene symbol is located in column A, log fold changes (logFC) in column B, average expression in column C, t-values in column D, P-value in column

E and adjusted P-values in column F. The raw data for the corresponding Gene Ontology enrichment analysis performed using Enrichr is provided in tab 2 (Biological processes) and tab 3 (Molecular functions). The raw data for the corresponding signaling pathways enrichment analysis performed using Enrichr is provided in tab 4 (reactome signaling pathways) and tab 5 (KEGG signaling pathways). For tabs 2-5, the terms, p-value, FDR, Z-score, combined score and corresponding genes are provided in columns A-F (up regulated), respectively, or columns I-N (down regulated), respectively. The top 15 terms (which are illustrated in the corresponding figure) are color-coded (red for up regulated or blue for down regulated) and terms that had p-values <0.05 and FDR<0.1 are bolded.

Suppl. Table S5D. Raw RNAseq/enrichment results for CCA Immune subtypes C2 v C3. The raw RNAseq data used to generate the differential expression analysis (volcano plot and corresponding table with CmPn expression differences) in Suppl. Fig 2D is provided in tab 1; gene symbol is located in column A, log fold changes (logFC) in column B, average expression in column C, t-values in column D, P-value in column E and adjusted P-values in column F. The raw data for the corresponding Gene Ontology enrichment analysis performed using Enrichr is provided in tab 2 (Biological processes) and tab 3 (Molecular functions). The raw data for the corresponding signaling pathways enrichment analysis performed using Enrichr is provided in tab 4 (reactome signaling pathways) and tab 5 (KEGG signaling pathways). For tabs 2-5, the terms, p-value, FDR, Z-score, combined score and corresponding genes are provided in columns A-F (up regulated), respectively, or columns I-N (down regulated), respectively. The top 15 terms (which are illustrated in the corresponding figure) are color-coded (red for up regulated or blue for down regulated) and terms that had p-values <0.05 and FDR<0.1 are bolded.

Suppl. Table S5E. Raw RNAseq/enrichment results for CCA Immune subtypes C2 v C4. The raw RNAseq data used to generate the differential expression analysis (volcano plot and corresponding table with

CmPn expression differences) in Suppl. Fig 2E is provided in tab 1; gene symbol is located in column A, log fold changes (logFC) in column B, average expression in column C, t-values in column D, P-value in column E and adjusted P-values in column F. The raw data for the corresponding Gene Ontology enrichment analysis performed using Enrichr is provided in tab 2 (Biological processes) and tab 3 (Molecular functions). The raw data for the corresponding signaling pathways enrichment analysis performed using Enrichr is provided in tab 4 (reactome signaling pathways) and tab 5 (KEGG signaling pathways). For tabs 2-5, the terms, p-value, FDR, Z-score, combined score and corresponding genes are provided in columns A-F (up regulated), respectively, or columns I-N (down regulated), respectively. The top 15 terms (which are illustrated in the corresponding figure) are color-coded (red for up regulated or blue for down regulated) and terms that had p-values <0.05 and FDR<0.1 are bolded.

Suppl. Table S5F. Raw RNAseq/enrichment results for CCA Immune subtypes C3 v C4. The raw RNAseq data used to generate the differential expression analysis (volcano plot and corresponding table with CmPn expression differences) in Suppl. Fig 2F is provided in tab 1; gene symbol is located in column A, log fold changes (logFC) in column B, average expression in column C, t-values in column D, P-value in column E and adjusted P-values in column F. The raw data for the corresponding Gene Ontology enrichment analysis performed using Enrichr is provided in tab 2 (Biological processes) and tab 3 (Molecular functions). The raw data for the corresponding signaling pathways enrichment analysis performed using Enrichr is provided in tab 4 (reactome signaling pathways) and tab 5 (KEGG signaling pathways). For tabs 2-5, the terms, p-value, FDR, Z-score, combined score and corresponding genes are provided in columns A-F (up regulated), respectively, or columns I-N (down regulated), respectively. The top 15 terms (which are illustrated in the corresponding figure) are color-coded (red for up regulated or blue for down regulated) and terms that had p-values <0.05 and FDR<0.1 are bolded.

S1A

Immunological Subtypes	Sample size
Wound Healing (C1)	22
IFN-γ dominant (C2)	45

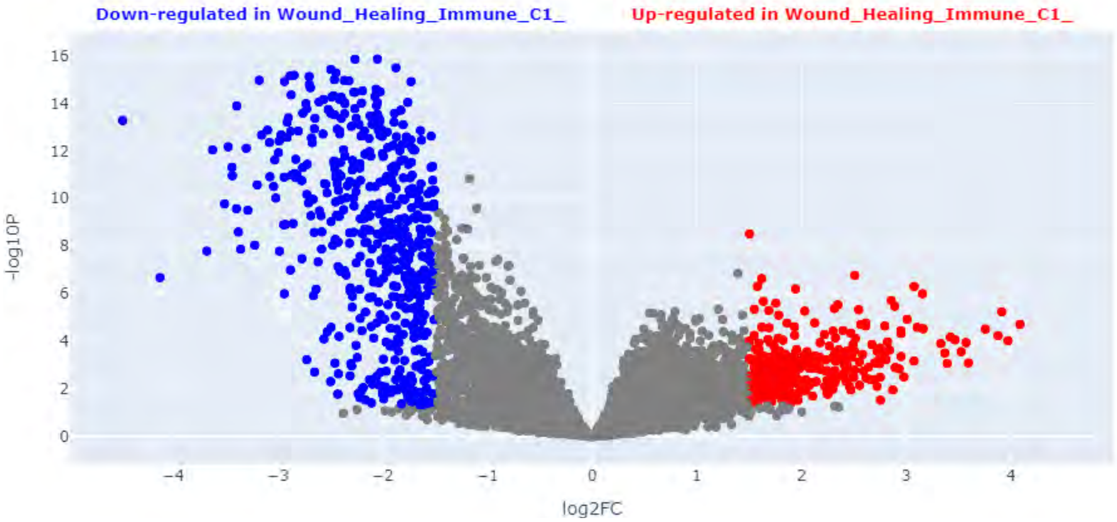

Gene symbol	logFC	AveExpr	t	P.Value
CCM1	0.518192	3.930383	3.14197	2.00E-03
PAQR6	0.68546	1.056312	2.055773	4.00E-02

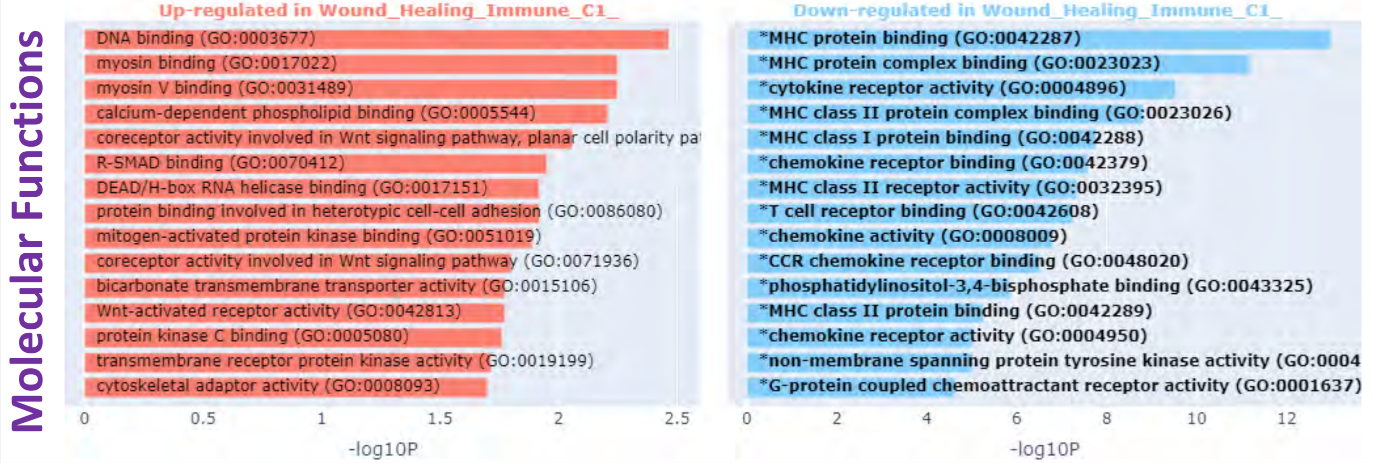

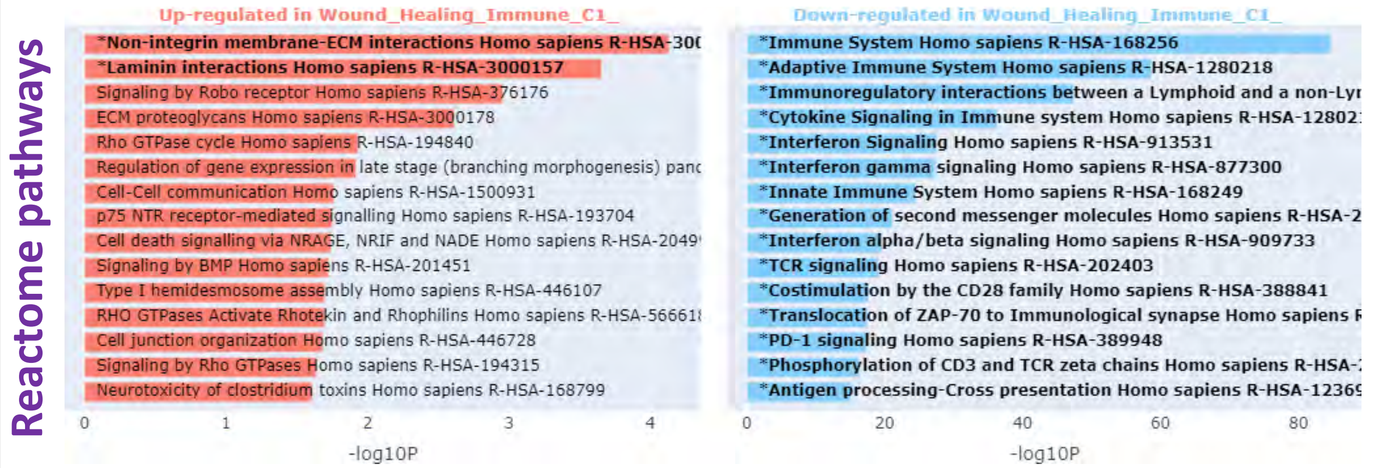

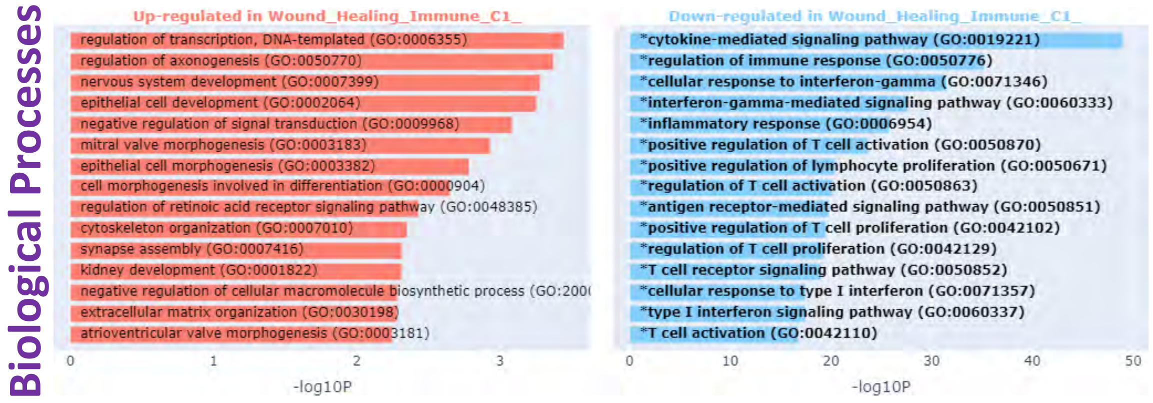

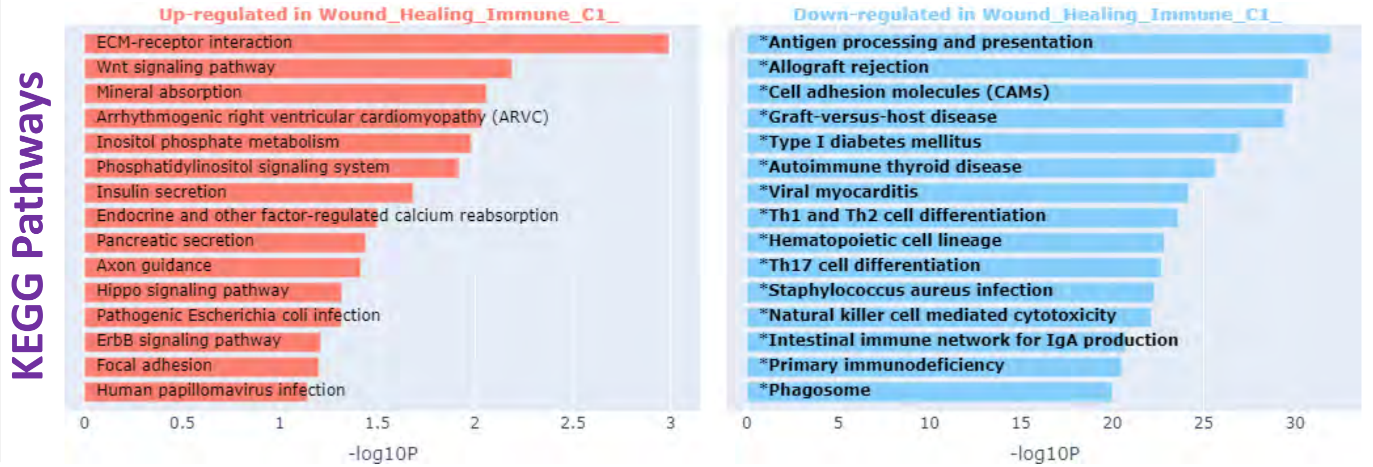

S1B

Immunological Subtypes	Sample size
Wound Healing (C1)	22
Inflammatory (C3)	135

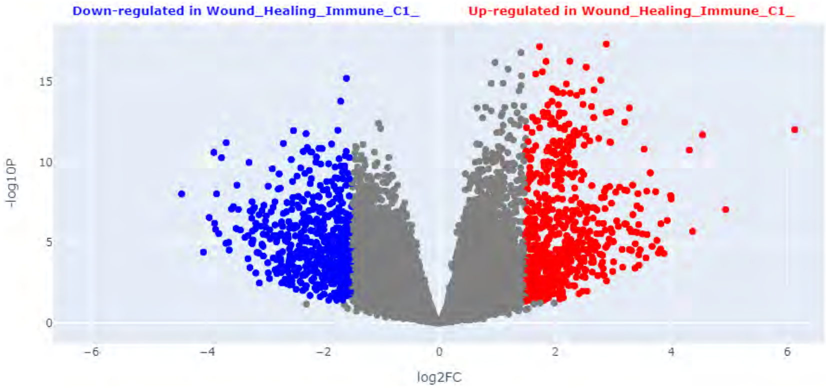

Gene symbol	logFC	AveExpr	t	P.Value
PAQR6	1.191942	0.344273	3.789672	2.14E-04
AFP	2.896023	3.312498	3.089215	2.37E-03
PAQR5	1.484179	2.100832	2.540652	1.20E-02
CCM3	0.200158	3.992807	2.209856	2.85E-02
PGRMC1	-1.14256	8.277952	-5.10998	9.15E-07
PGRMC2	-0.49476	6.458132	-3.84915	1.71E-04

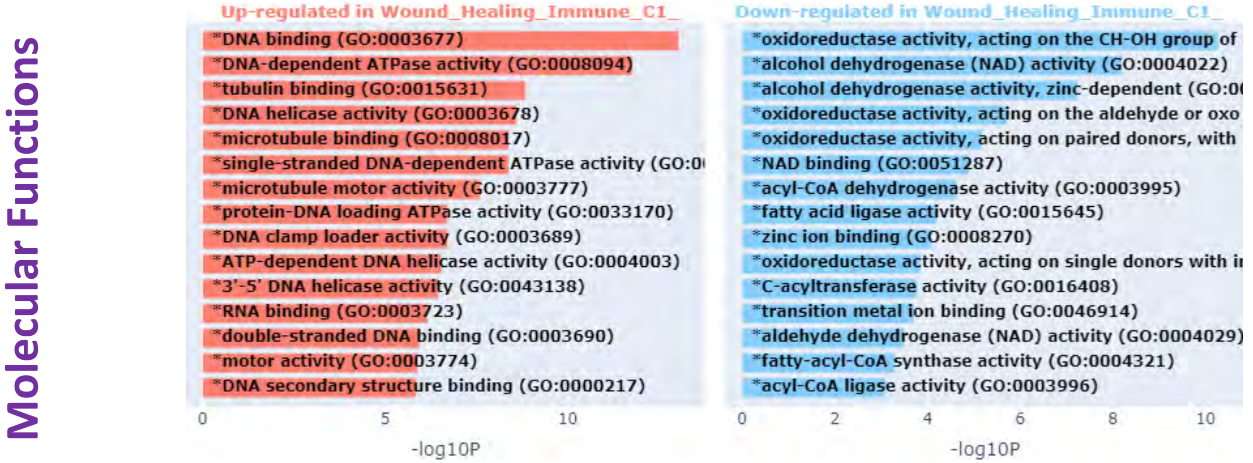

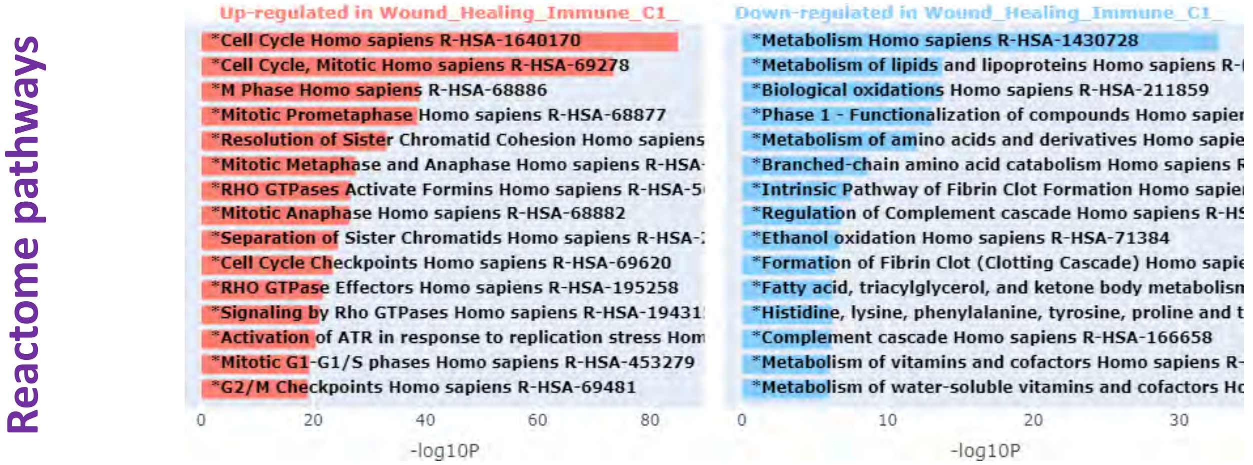

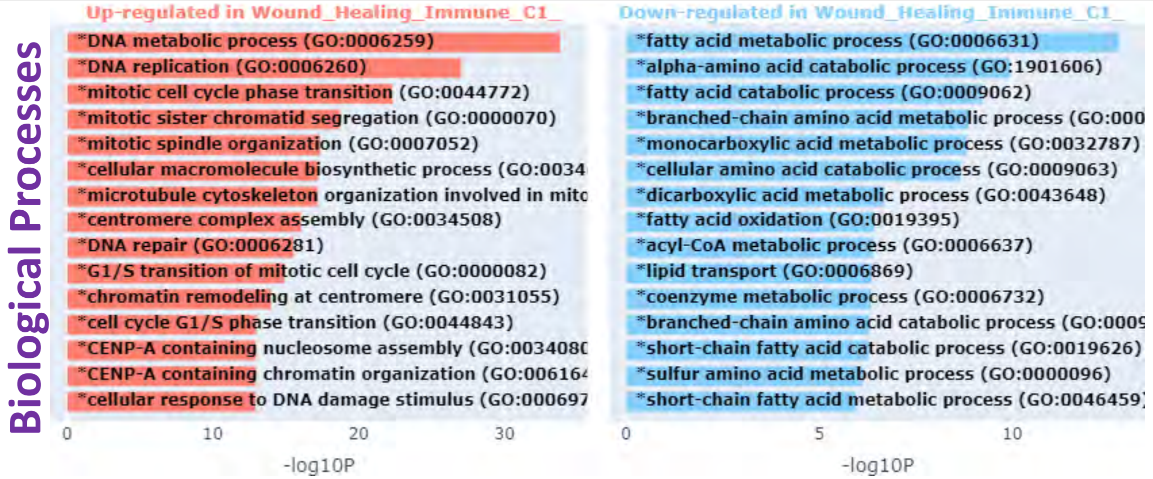

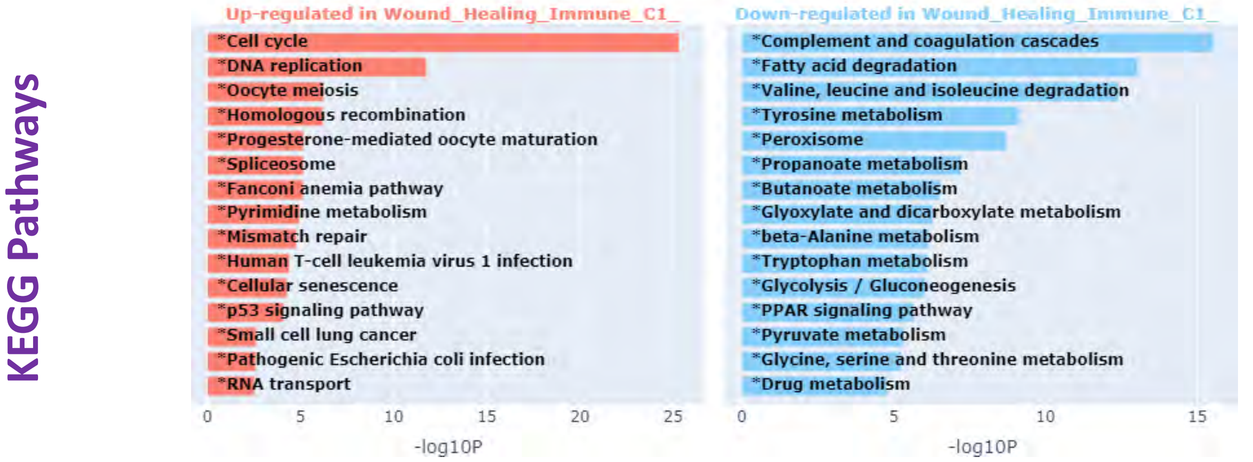

S1C

Immunological Subtypes	Sample size
Wound Healing (C1)	22
Lymphocyte depleted (C4)	159

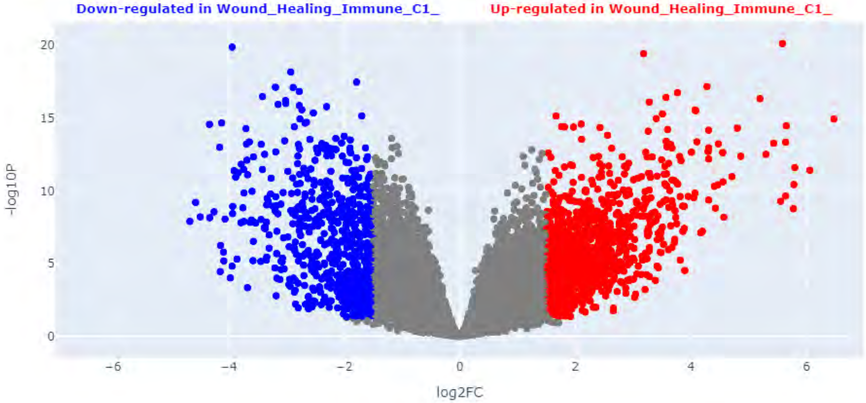

Gene symbol	logFC	AveExpr	t	P.Value
AFP	2.882463	3.183769	3.563241	**4.67E-04**
PAQR8	1.154065	1.557629	3.544084	**5.00E-04**
PAQR5	2.228739	1.326255	3.43325	**7.37E-04**
PAQR6	1.054751	0.347308	3.42513	**7.58E-04**
PGRMC1	-1.33634	8.378224	-6.01506	**9.56E-09**
PGRMC2	-0.64401	6.503715	-5.01827	**1.23E-06**
PAQR9	-0.8919	4.253548	-3.13713	**1.99E-03**
CCM2	-0.3834	6.047957	-2.40294	**1.73E-02**

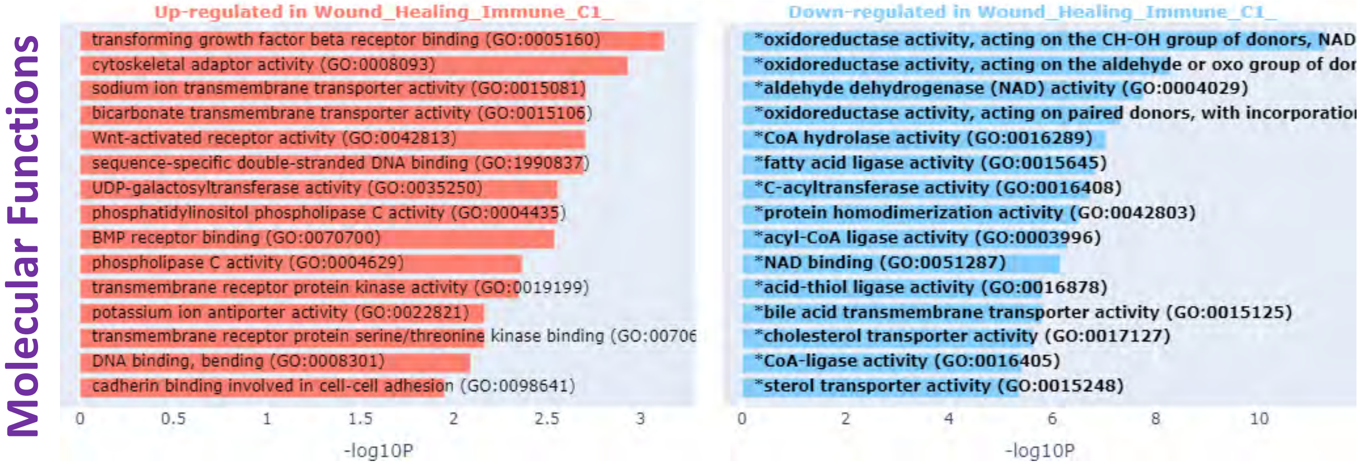

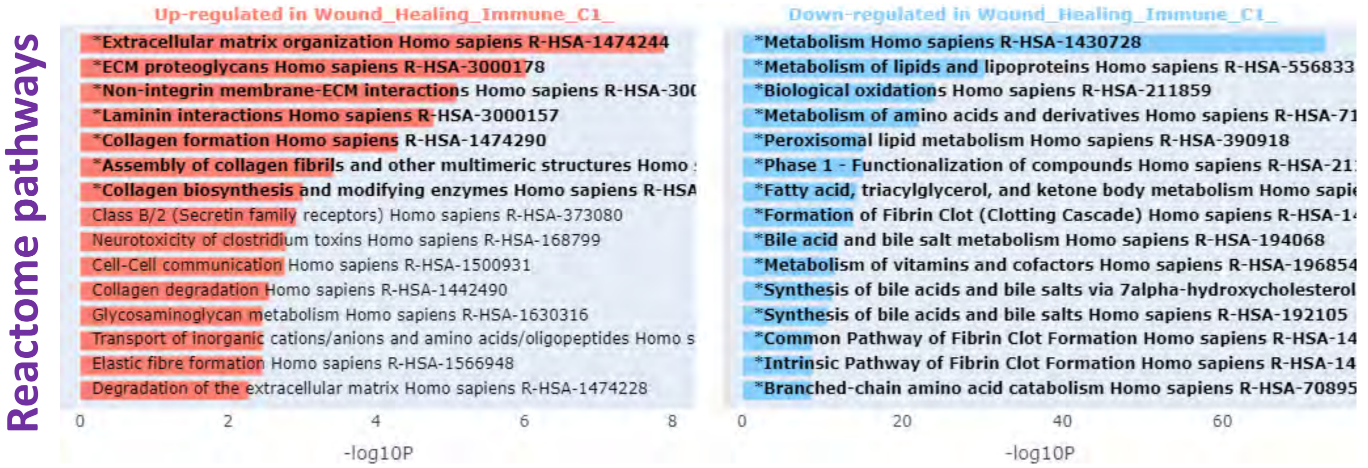

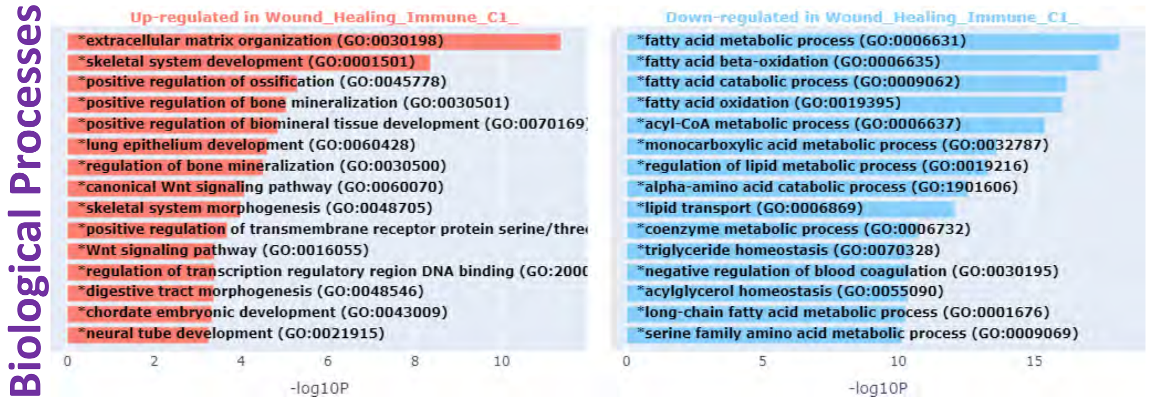

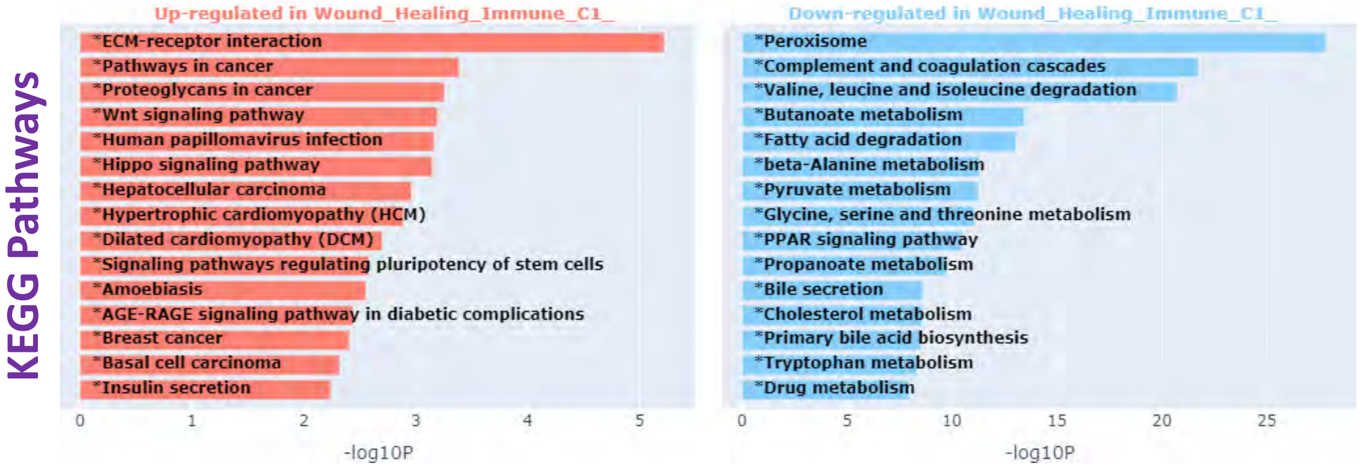

S1D

Immunological Subtypes	Sample size
IFN-γ dominant (C2)	45
Inflammatory (C3)	135

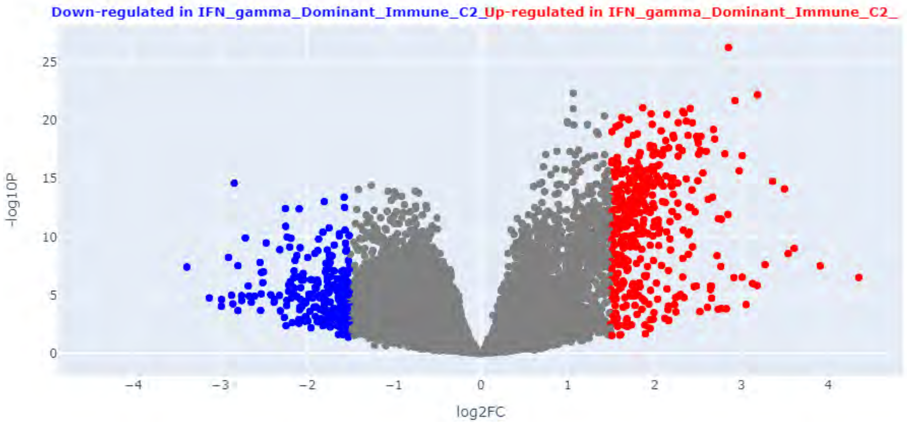

Gene symbol	logFC	AveExpr	t	P.Value
AFP	3.916882	3.872694	5.782277	3.16E-08
CCM3	0.153459	3.986799	2.163984	3.18E-02
PAQR6	0.500112	0.287465	2.111144	3.61E-02
PAQR8	0.42792	2.253244	2.005111	4.64E-02
PGRMC1	-0.7814	8.228141	-4.66774	5.90E-06
CCM1	-0.47107	3.94761	-4.57818	8.68E-06
nPRs	-0.98985	-1.40387	-2.97771	3.30E-03
PGRMC2	-0.29999	6.436494	-2.93692	3.74E-03

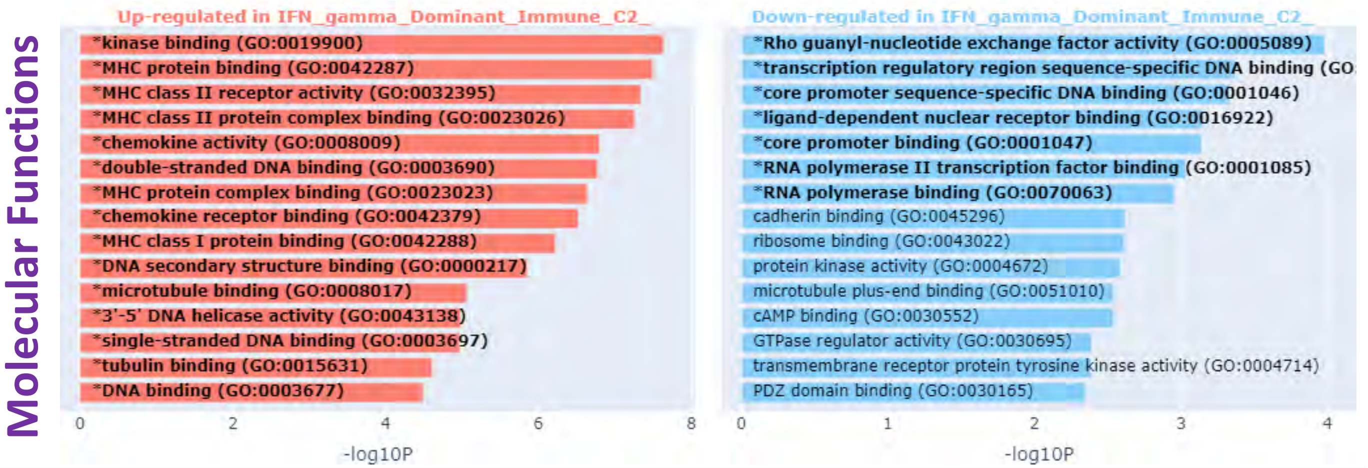

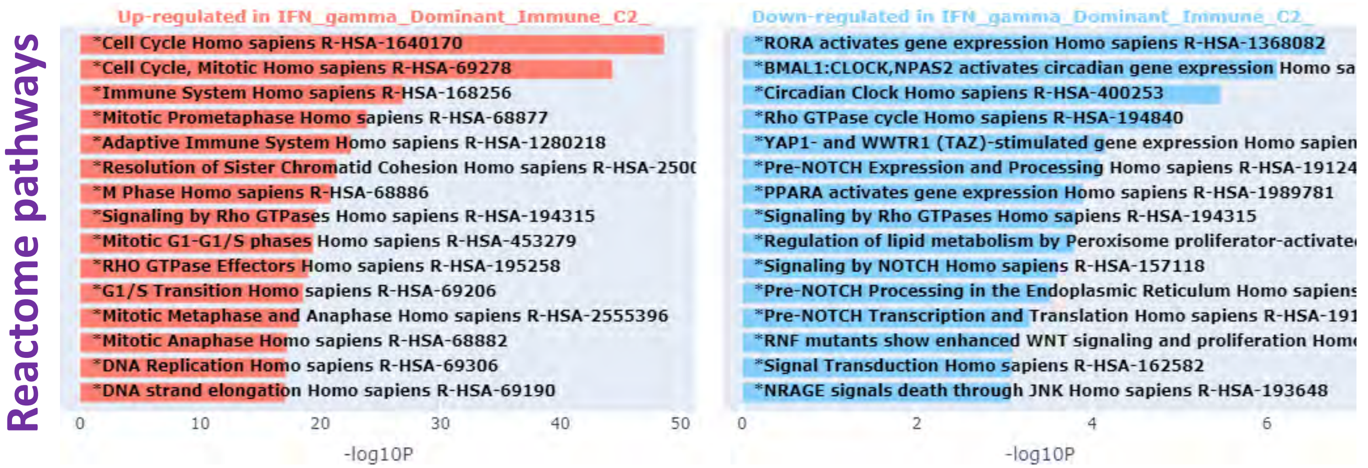

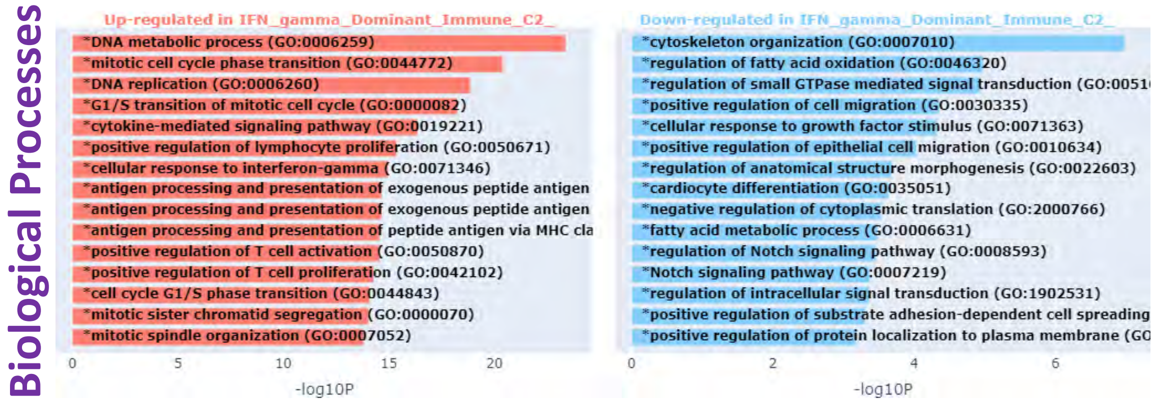

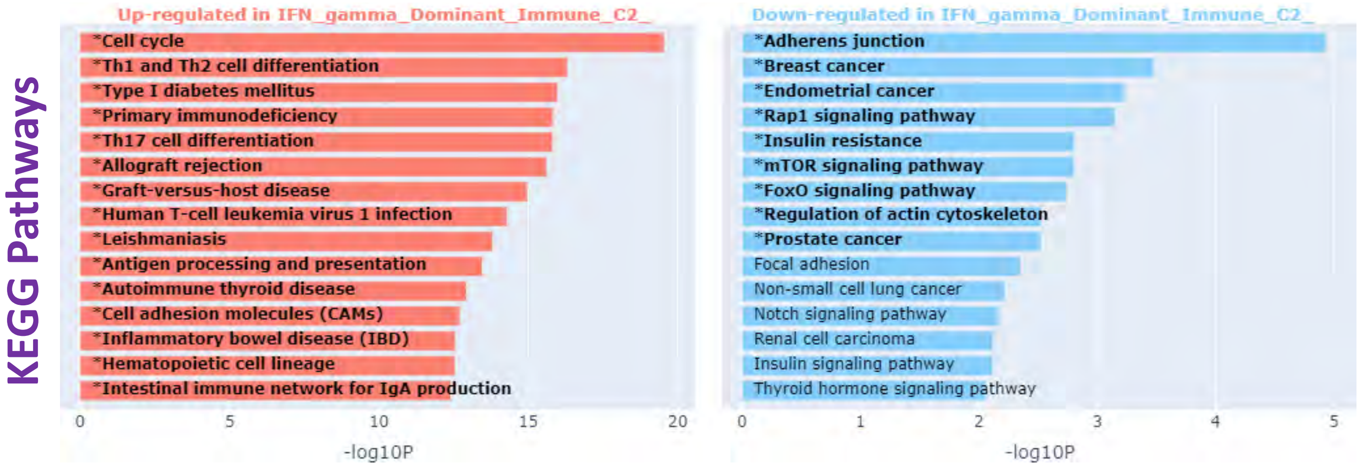

S1E

Immunological Subtypes	Sample size
IFN-γ dominant (C2)	45
Lymphocyte depleted (C4)	159

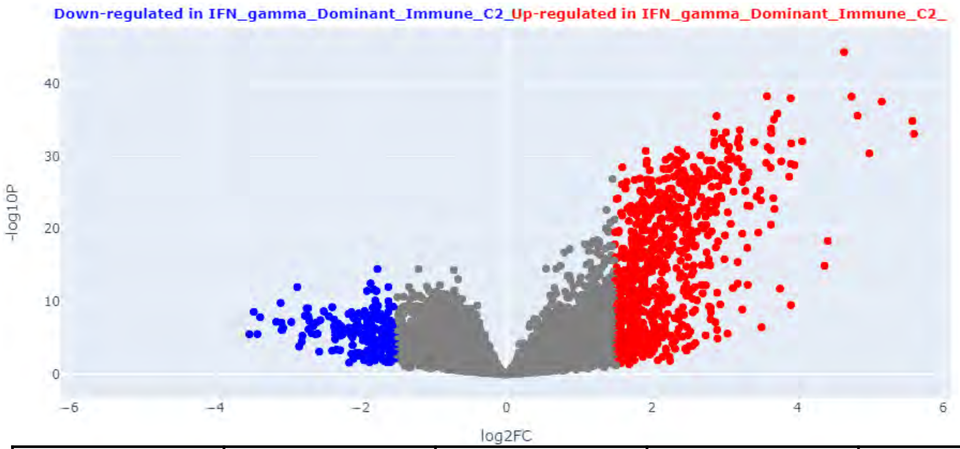

Gene symbol	logFC	AveExpr	t	P.Value
AFP	3.921464	3.692572	6.630822	2.88E-10
PAQR8	1.116469	1.652463	4.713229	4.48E-06
PAQR5	1.129452	1.295504	2.361378	1.91E-02
CCM1	-0.37457	3.833406	-3.77653	2.08E-04
PGRMC1	-0.9364	8.322968	-5.7336	3.47E-08
PGRMC2	-0.40891	6.479483	-4.12465	5.39E-05

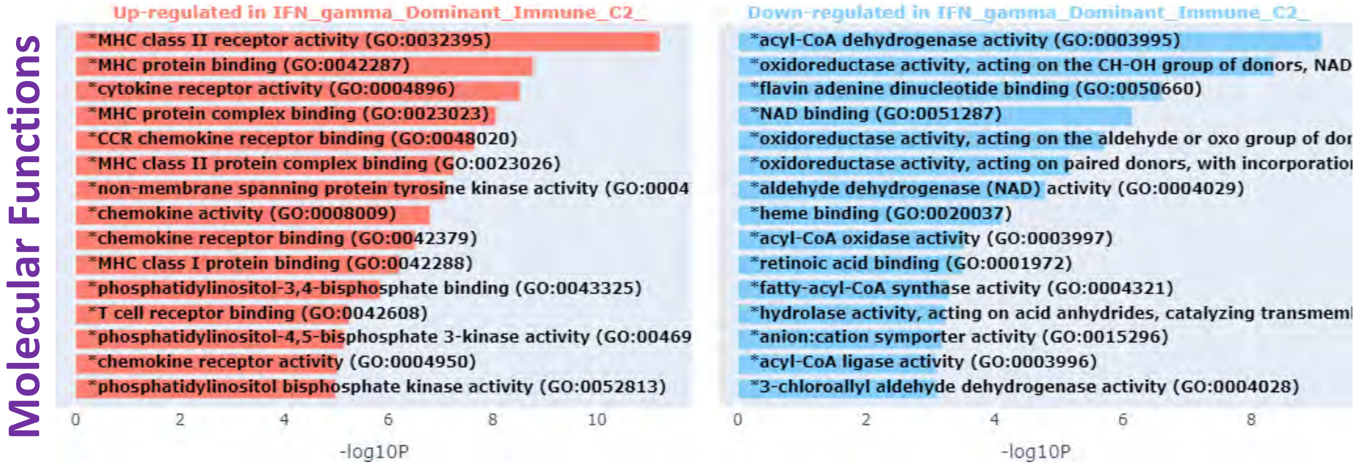

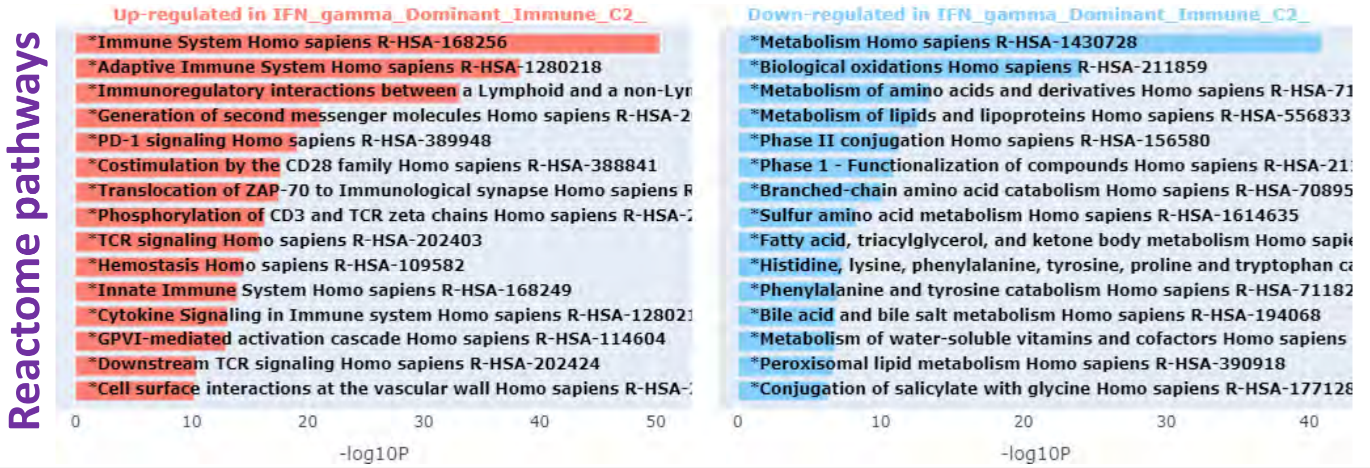

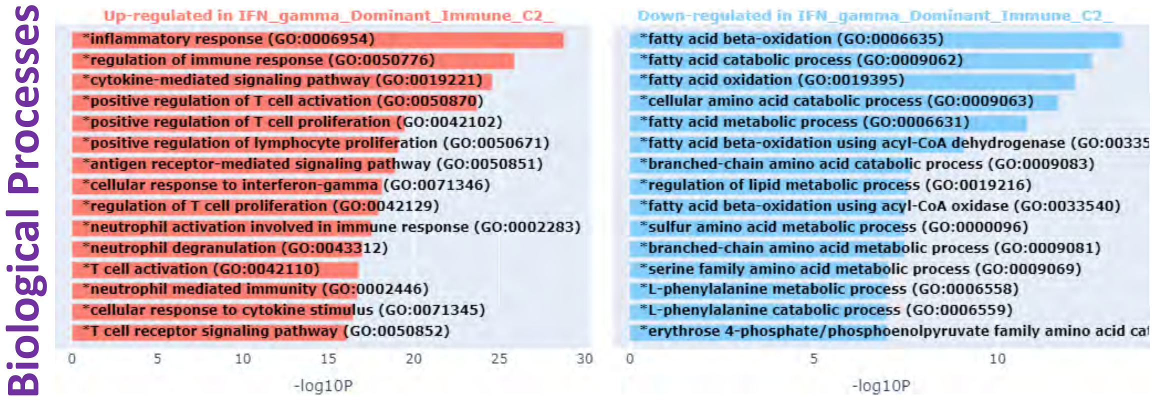

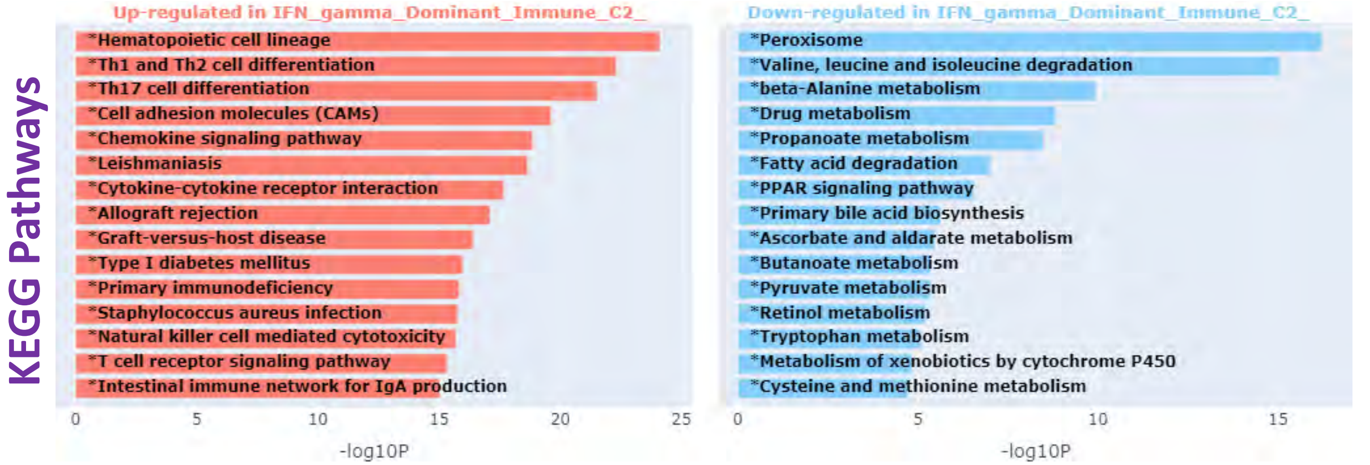

S1F

Immunological Subtypes	Sample size
Inflammatory (C3)	135
Lymphocyte depleted (C4)	159

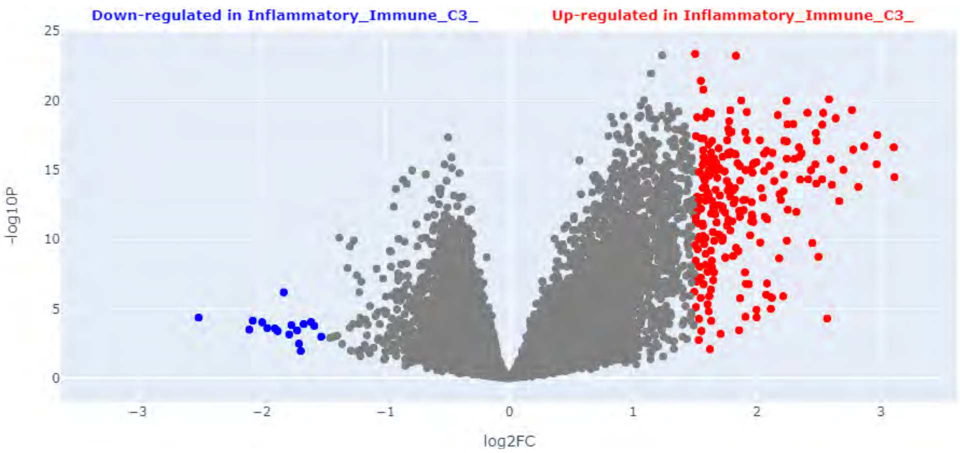

Gene symbol	logFC	AveExpr	t	P.Value
nPRs	1.510682	-2.04635	6.092938	3.45E-09
PAQR8	0.684746	1.709085	4.151653	4.32E-05
PAQR5	0.776807	1.385898	2.33205	2.04E-02
CCM2	-0.20795	5.975757	-2.57951	1.04E-02
CCM3	-0.1836	3.980731	-3.29313	1.11E-03

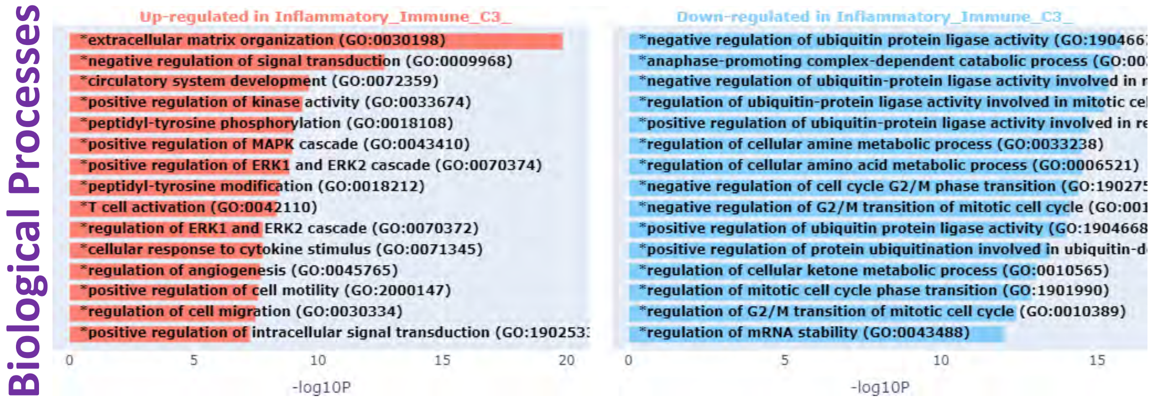

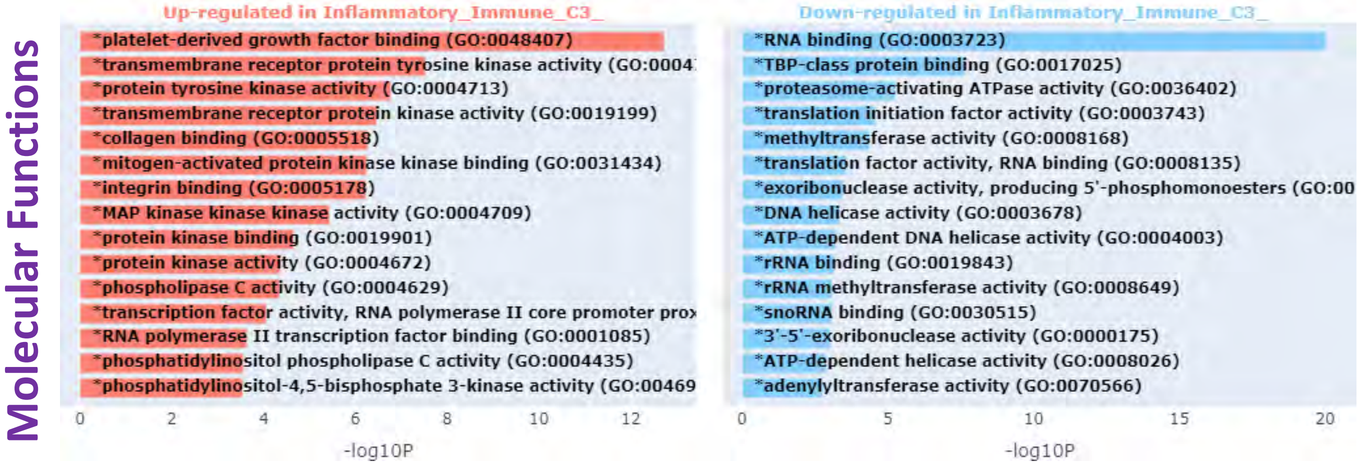

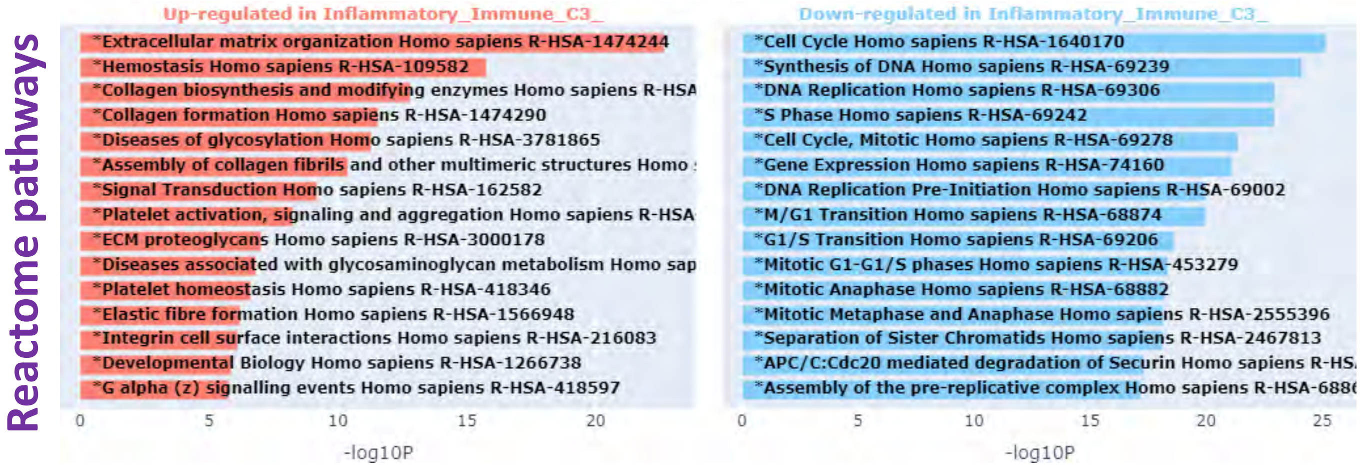

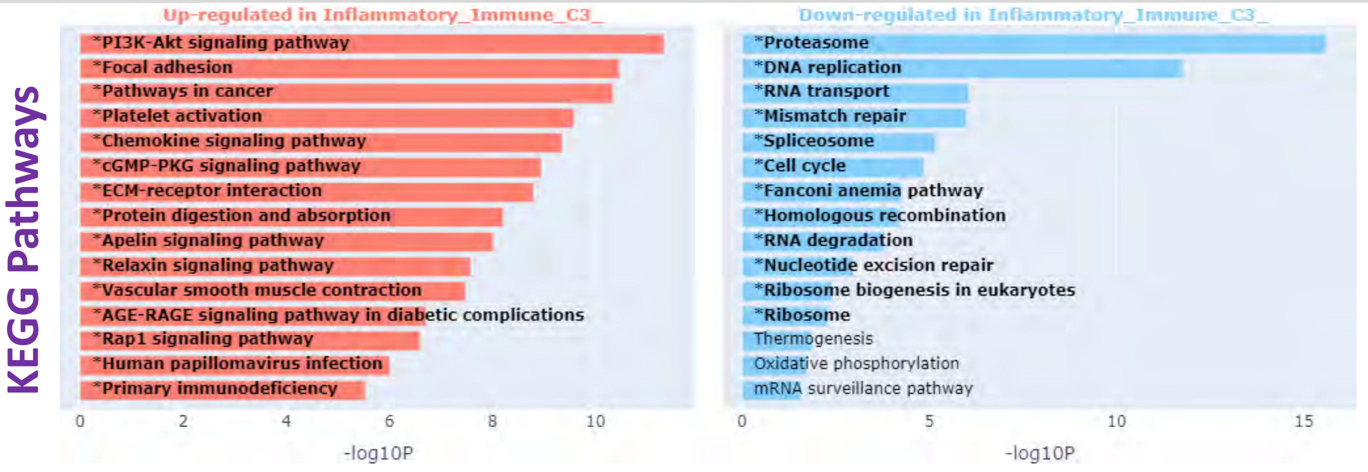

S2A

Immunological Subtypes	Sample size
Wound Healing (C1)	7
IFN-γ dominant (C2)	2

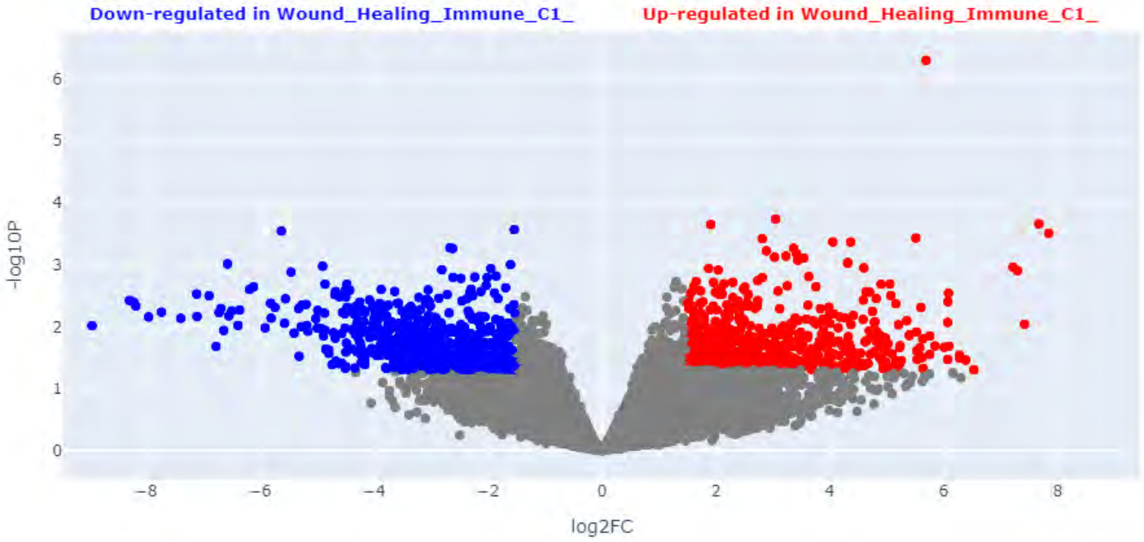

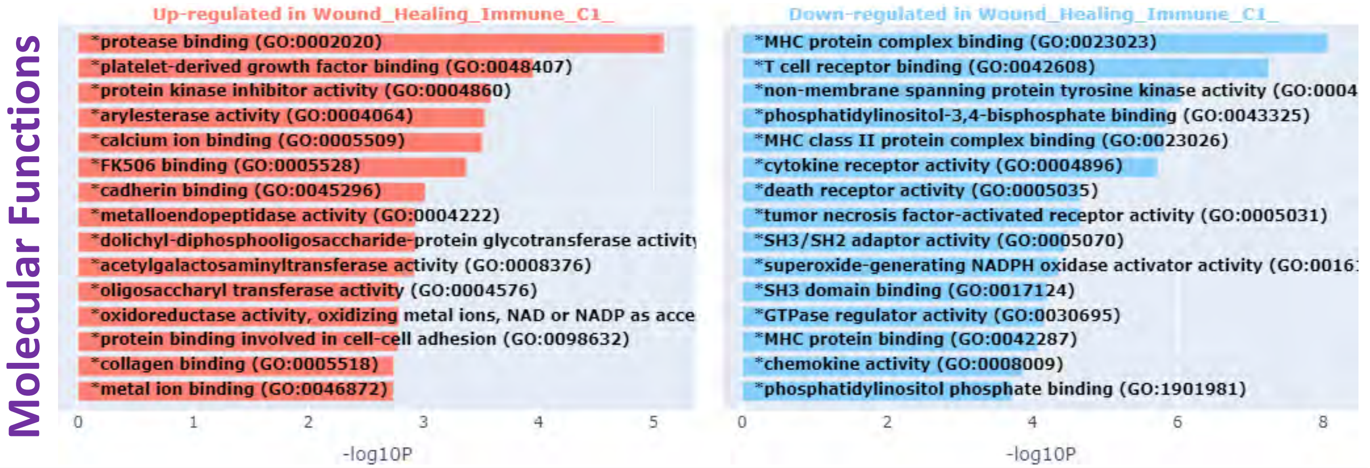

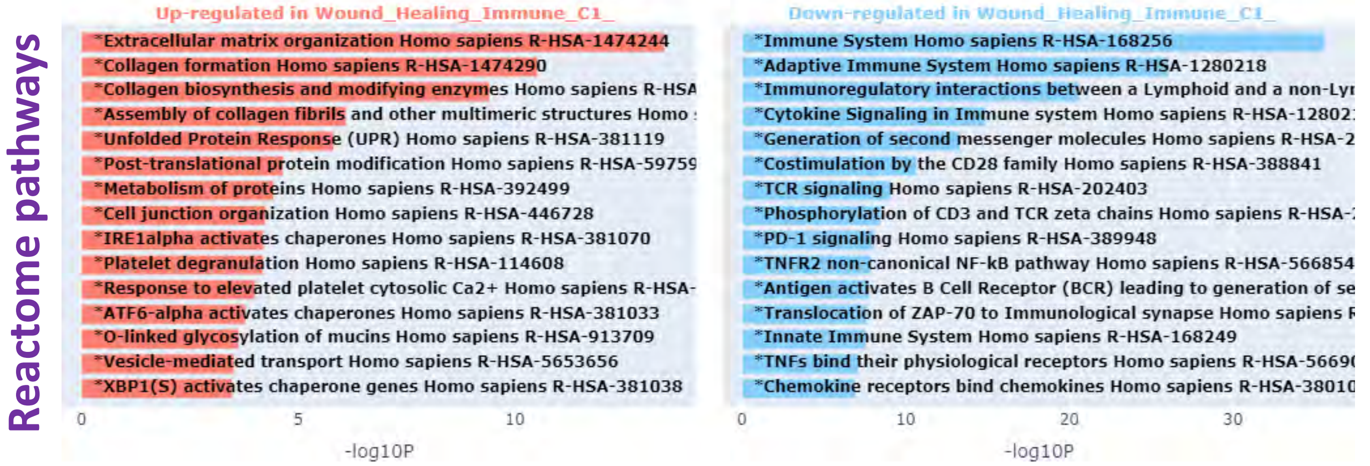

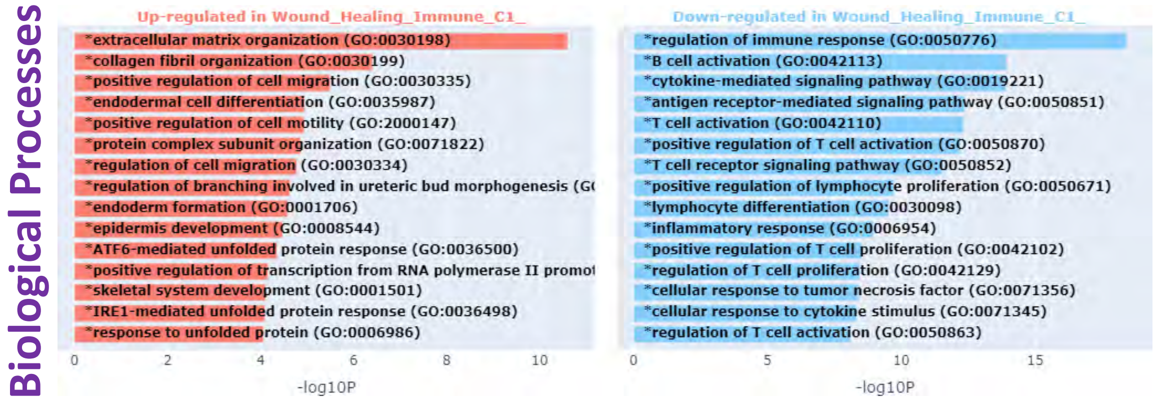

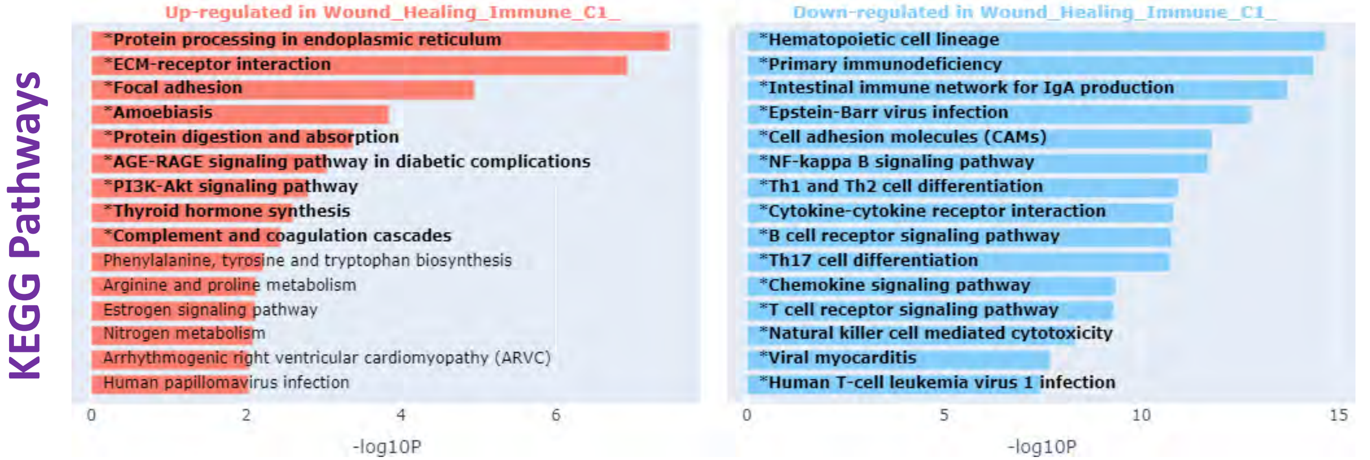

S2B

Immunological Subtypes	Sample size
Wound Healing (C1)	7
Inflammatory (C3)	17

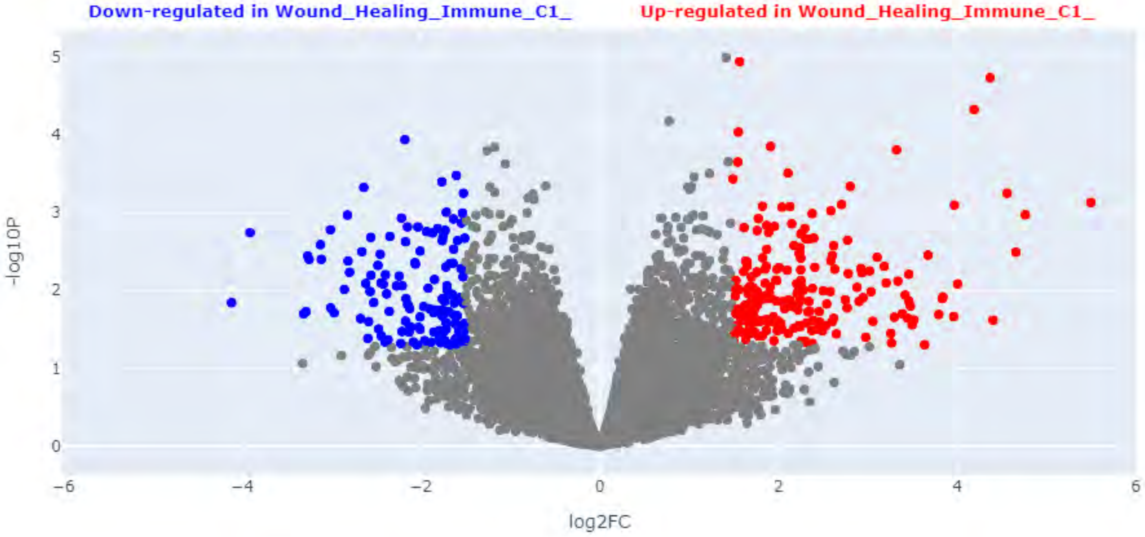

Gene symbol	logFC	AveExpr	t	P.Value
PAQR6	-1.3678	2.986596	-2.77353	1.02E-02

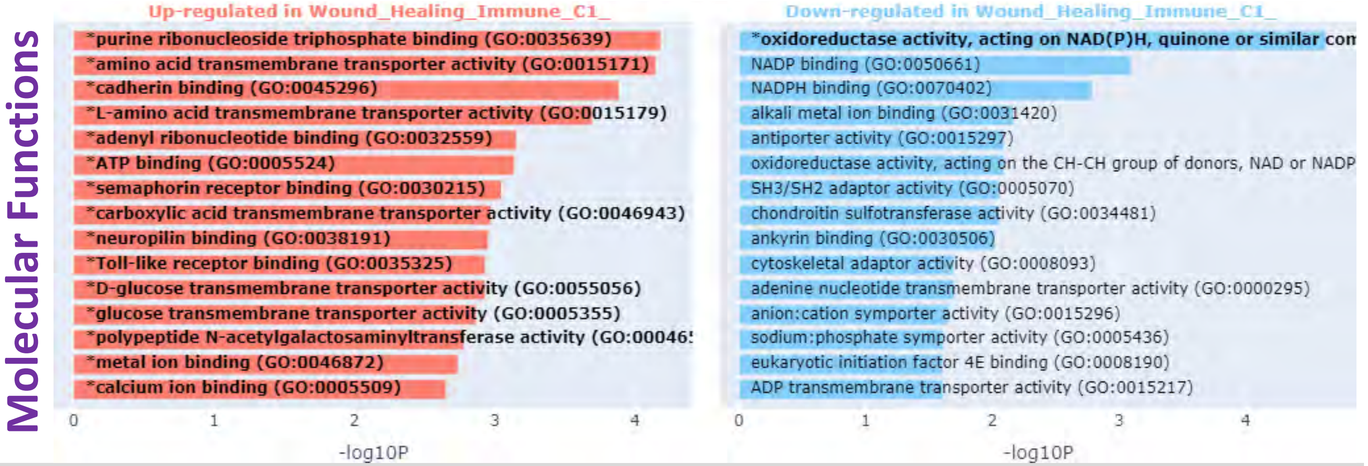

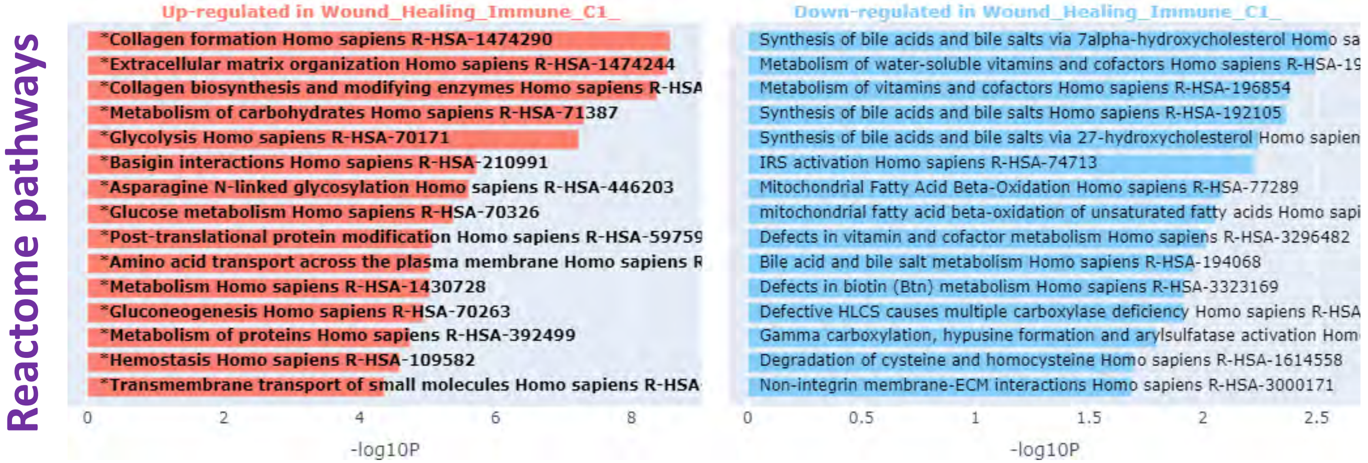

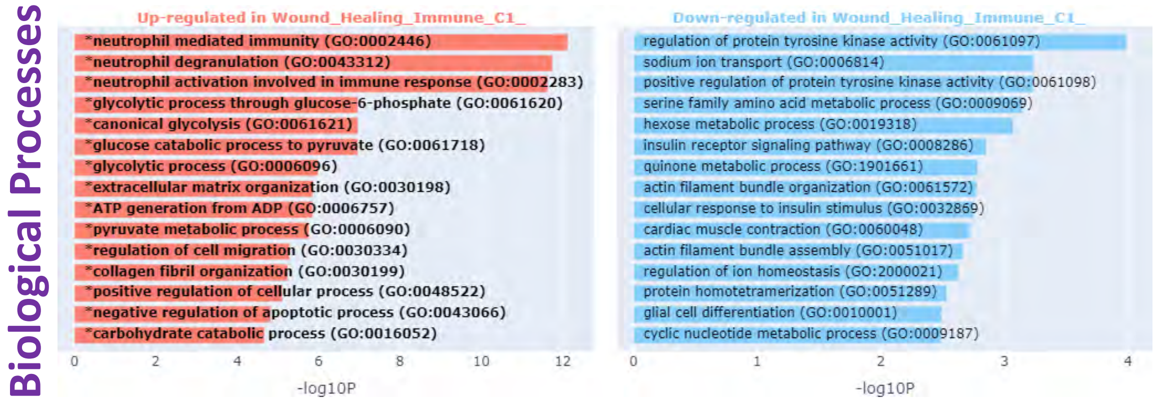

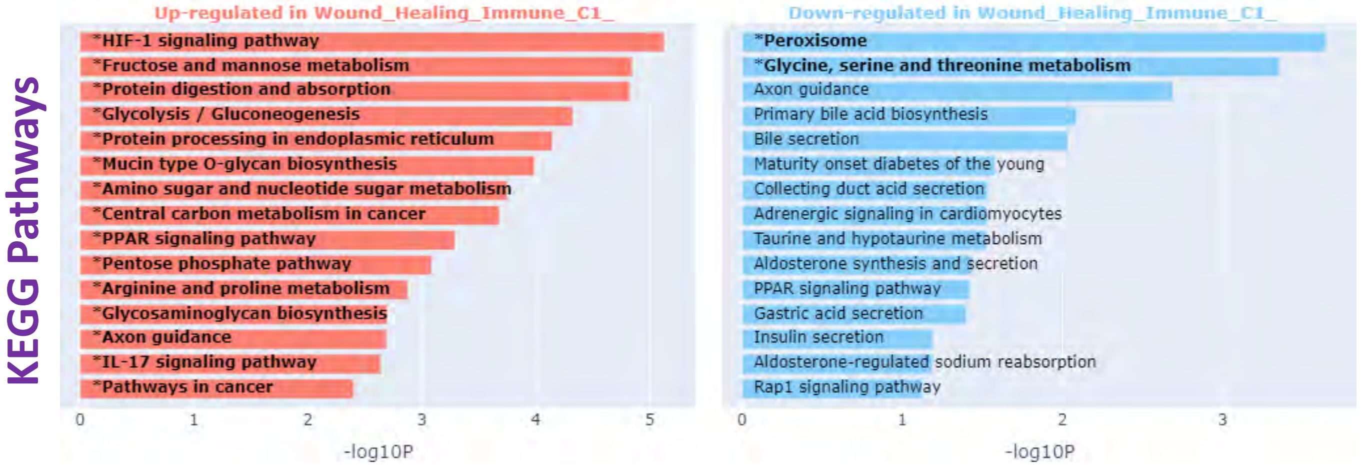

S2C

Immunological Subtypes	Sample size
Wound Healing (C1)	7
Lymphocyte depleted (C4)	8

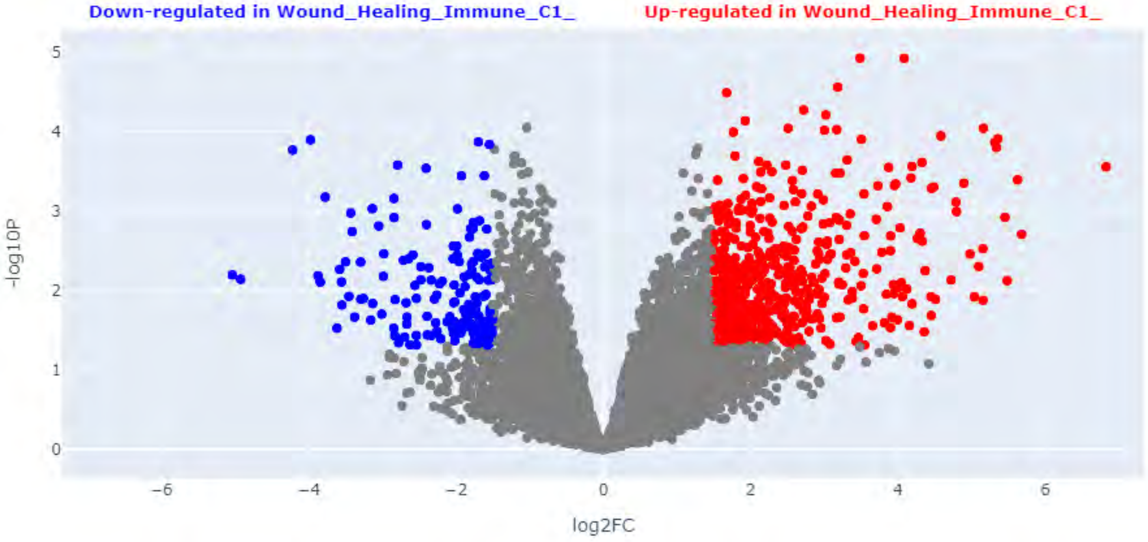

Gene symbol	logFC	AveExpr	t	P.Value
PAQR6	-1.47789	2.709641	-2.59804	1.90E-02

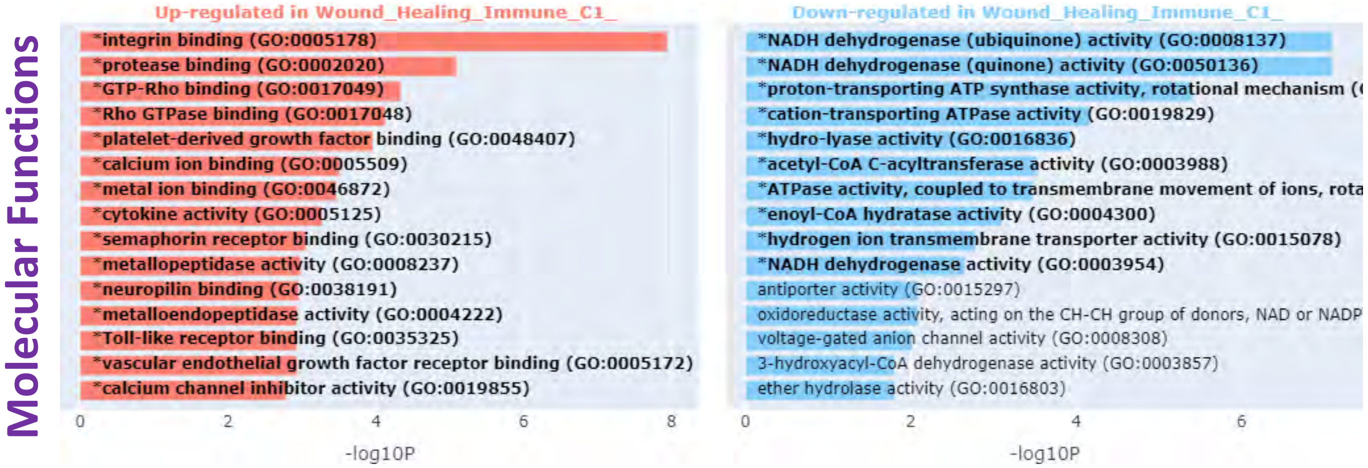

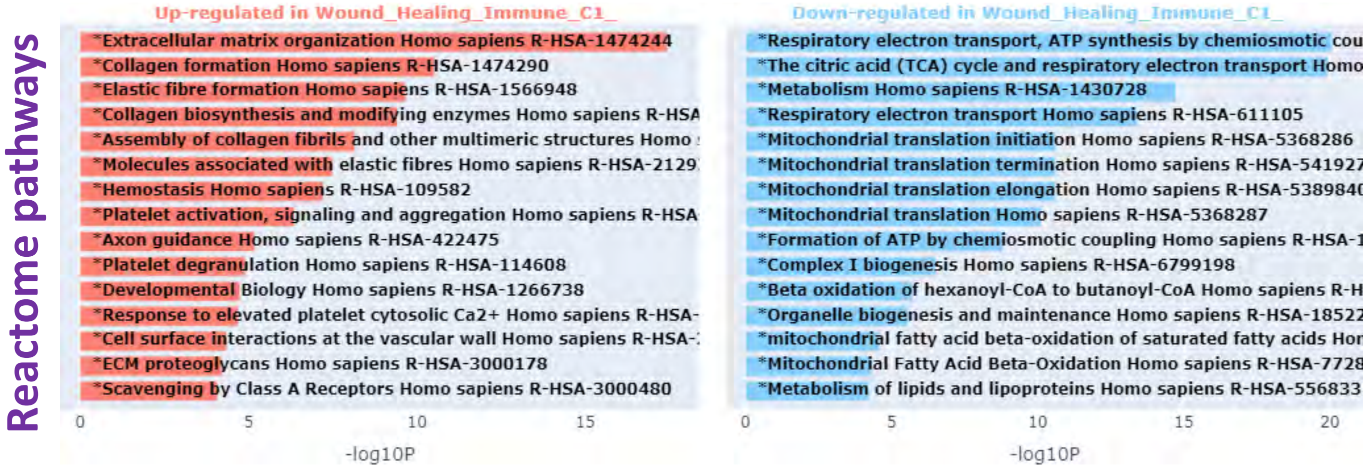

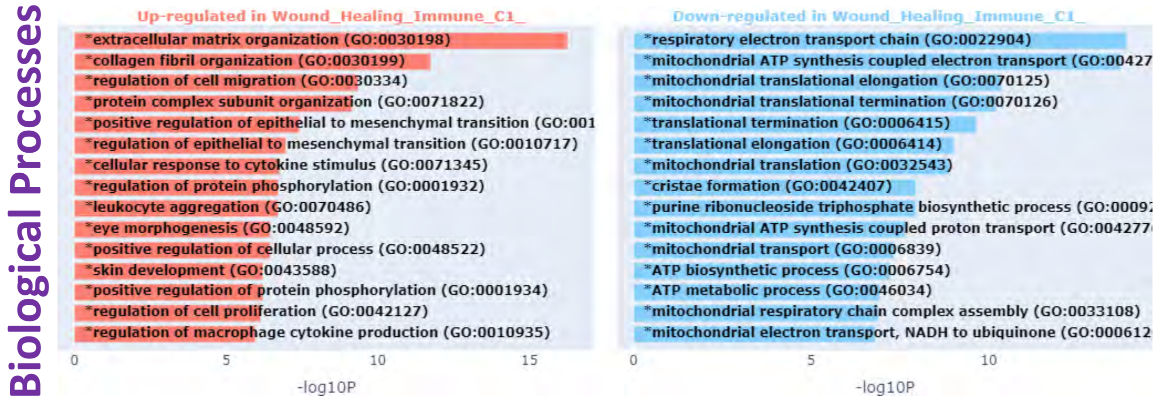

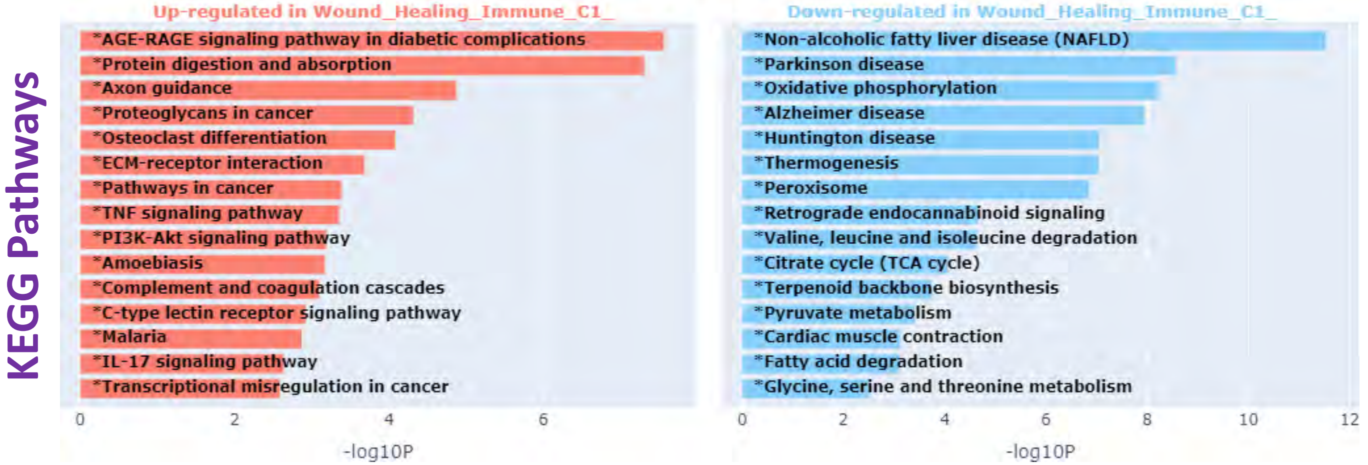

S2D

Immunological Subtypes	Sample size
IFN-γ dominant (C2)	2
Inflammatory (C3)	17

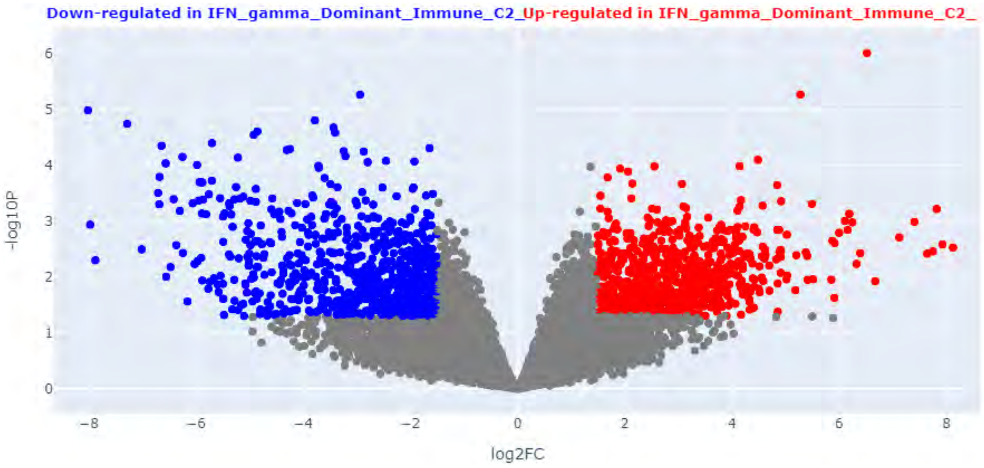

Gene symbol	logFC	AveExpr	t	P.Value
CCM2	1.031555	5.767136	2.317519	3.08E-02
PAQR6	-1.93451	3.191659	-2.32825	3.01E-02
PGRMC1	-1.08375	6.999647	-2.33136	2.99E-02
PAQR5	-3.83949	5.661036	-2.91322	8.40E-03

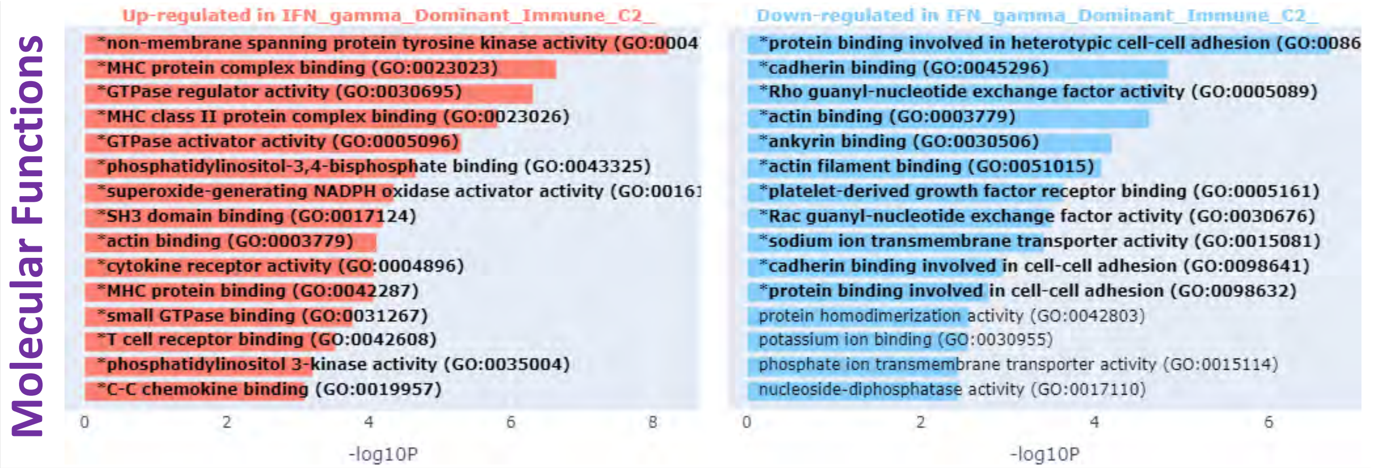

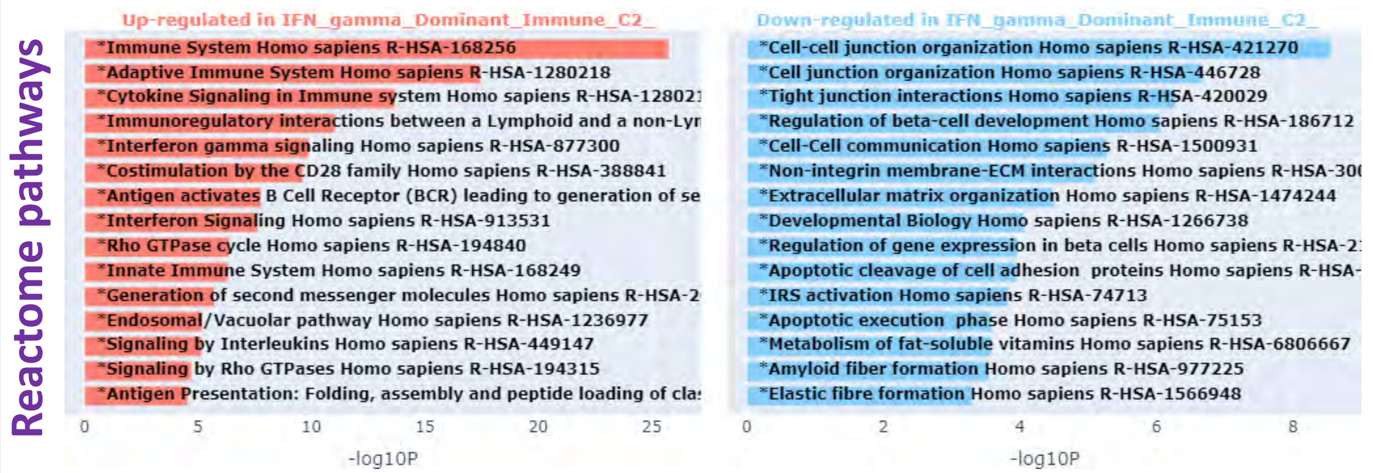

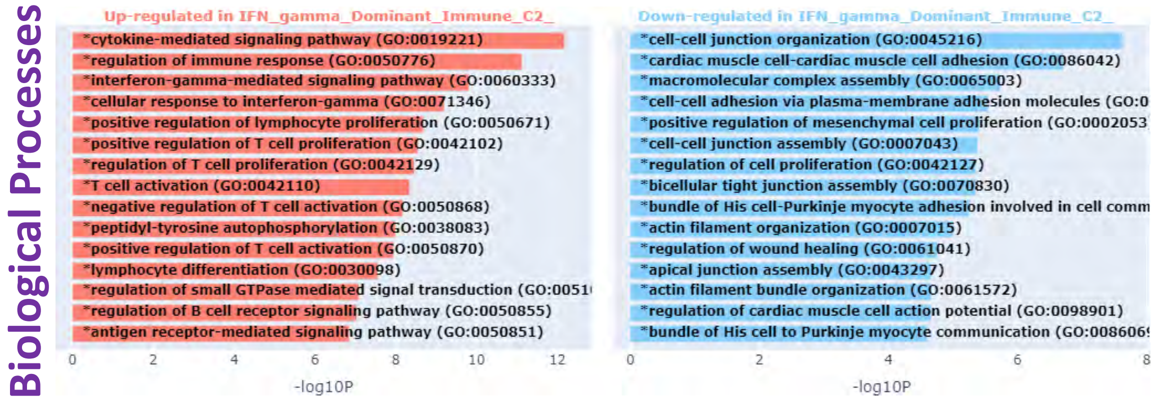

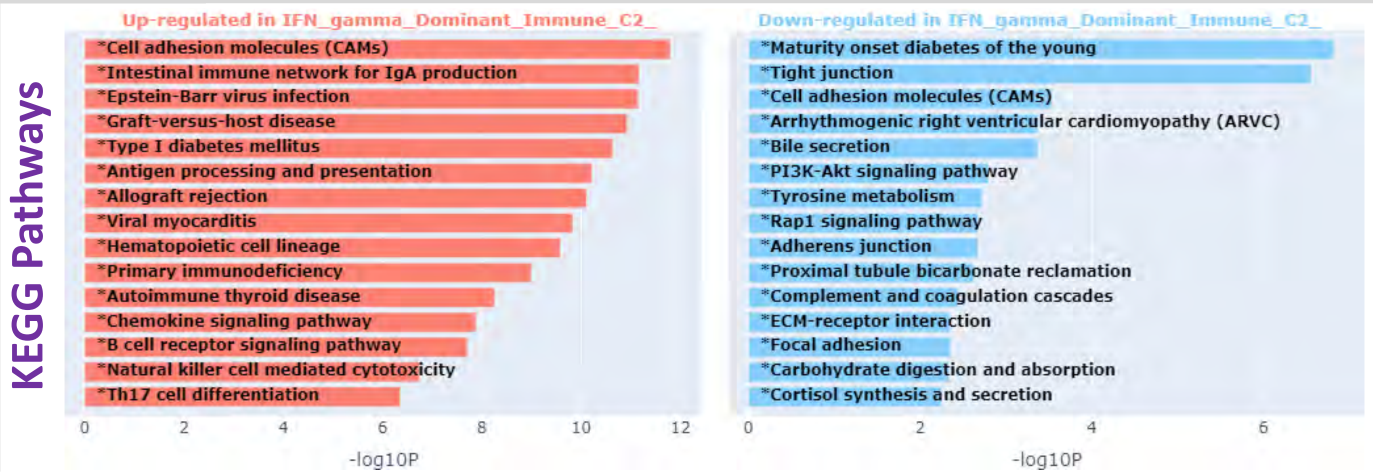

S2E

Immunological Subtypes	Sample size
IFN-γ dominant (C2)	2
Lymphocyte depleted (C4)	8

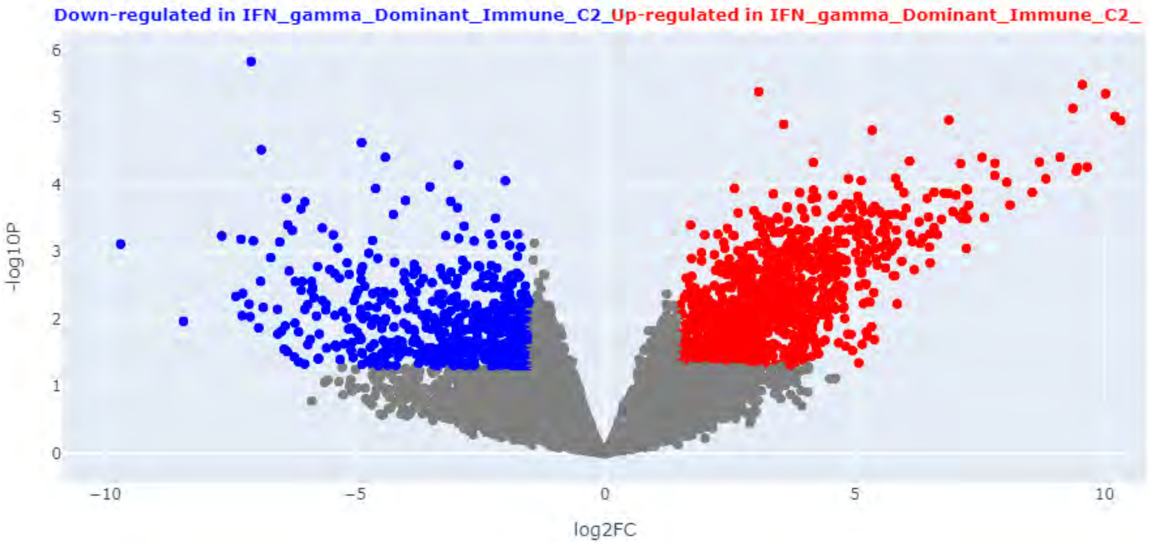

Gene symbol	logFC	AveExpr	t	P.Value
PGRMC1	-1.49332	7.052058	-2.27509	4.27E-02
PAQR6	-2.11244	2.960783	-2.41878	3.30E-02

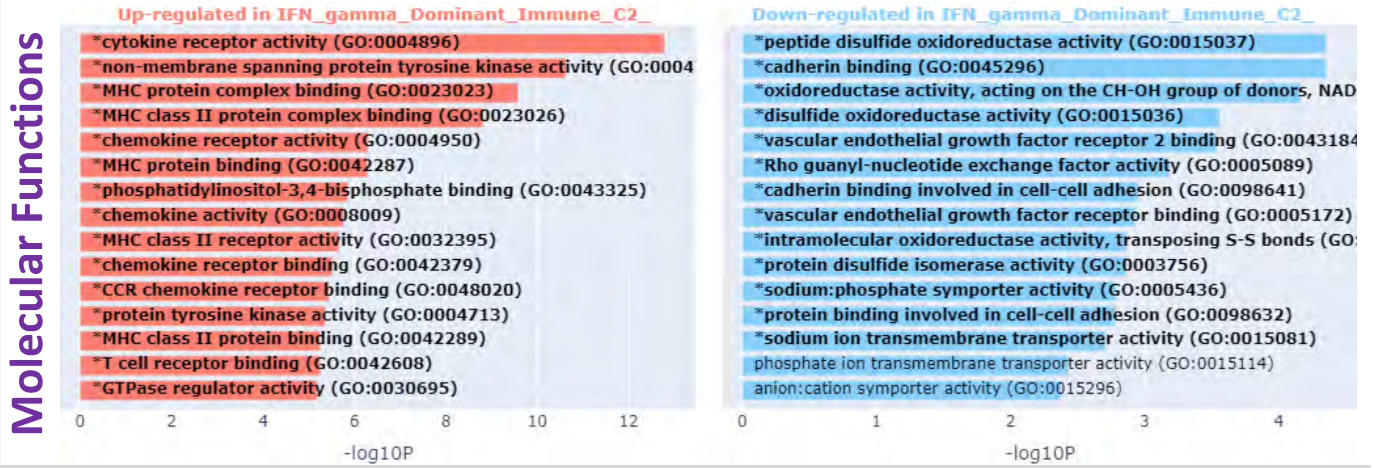

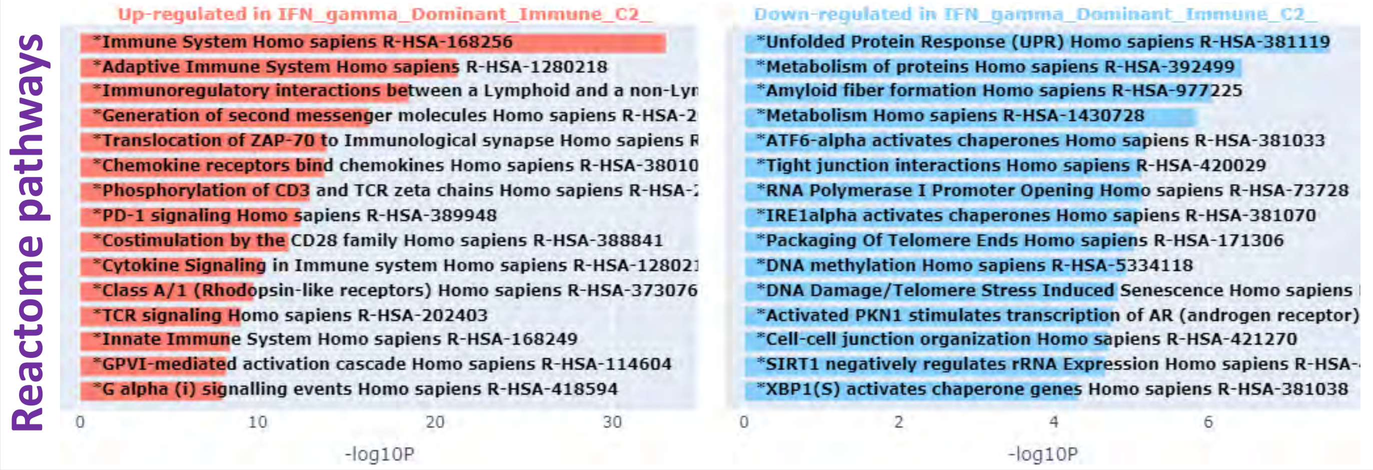

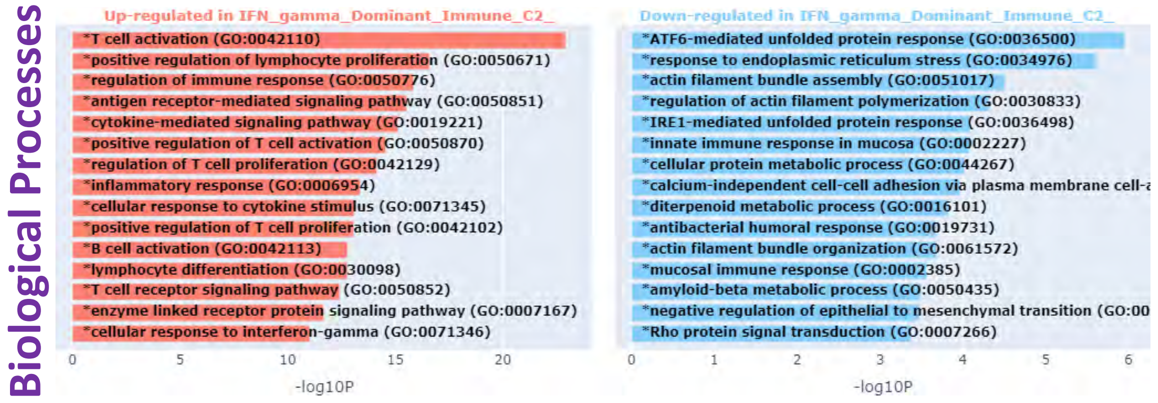

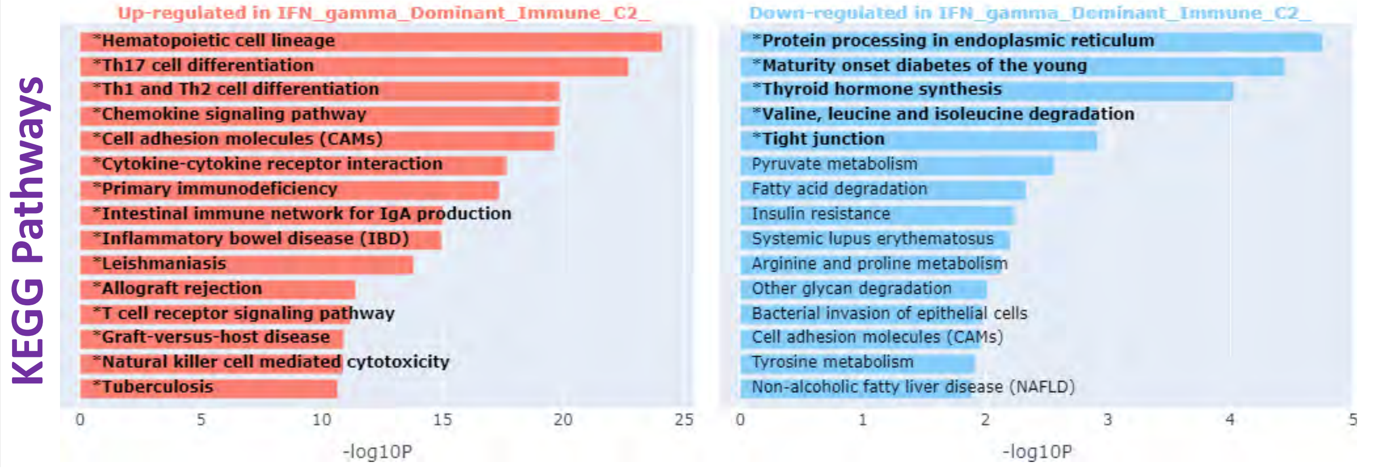

S2F

Immunological Subtypes	Sample size
Inflammatory (C3)	17
Lymphocyte depleted (C4)	8

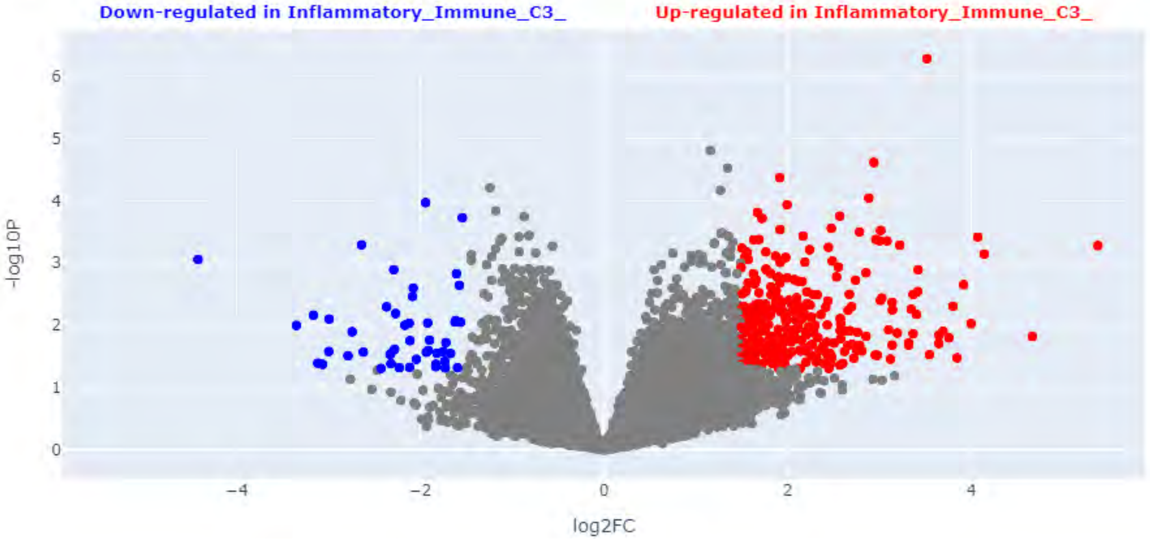

Gene symbol	logFC	AveExpr	t	P.Value
CCM3	-0.56277	4.842558	-2.64929	1.34E-02

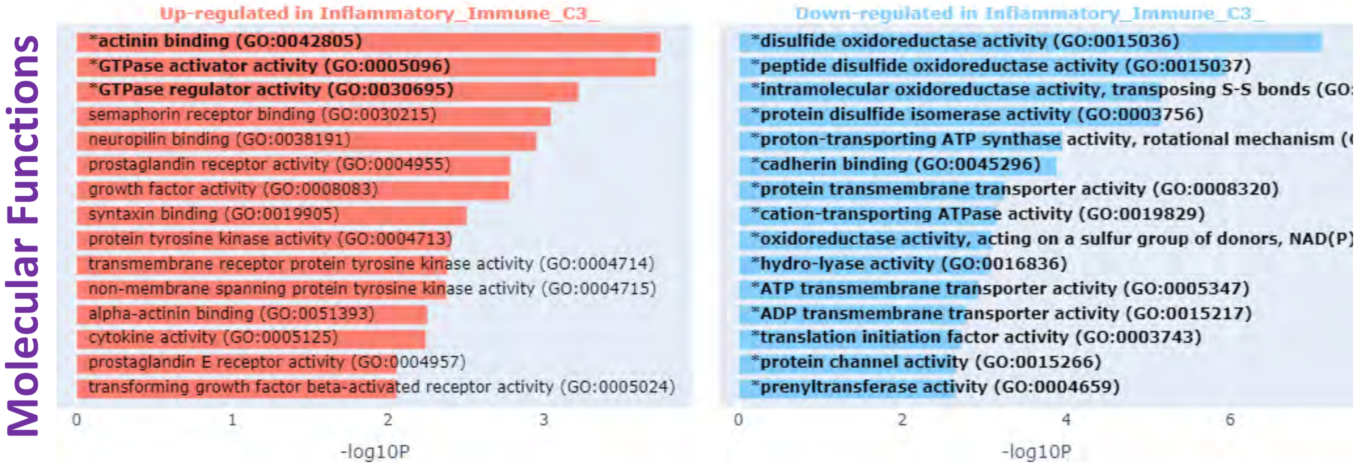

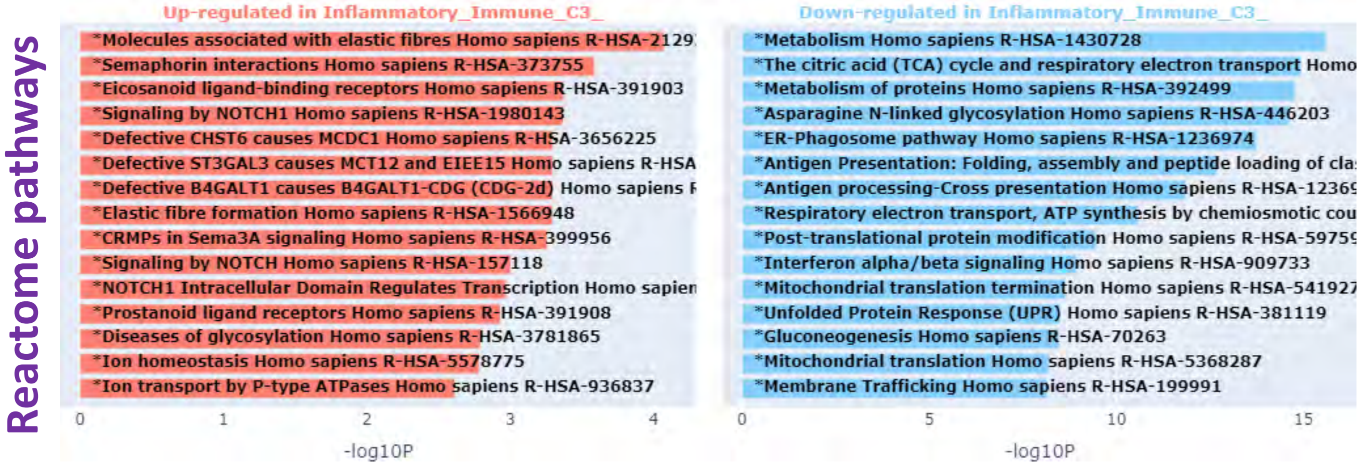

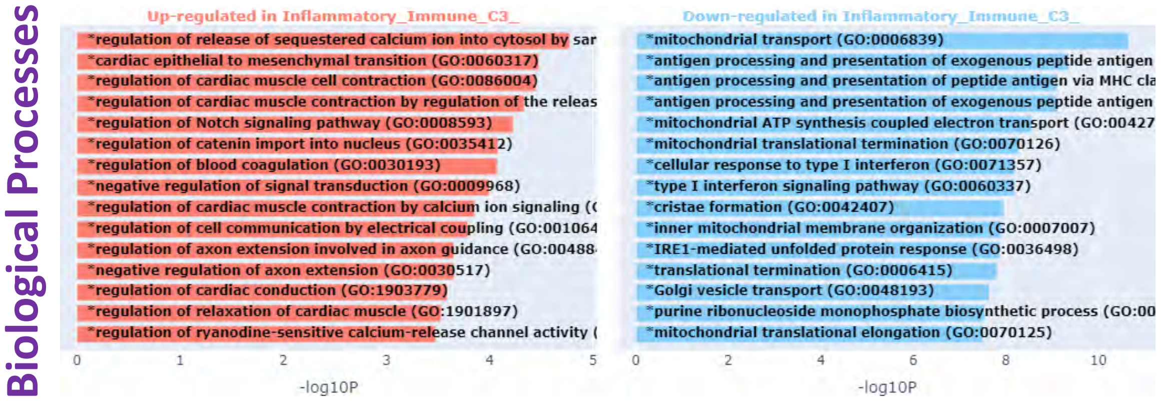

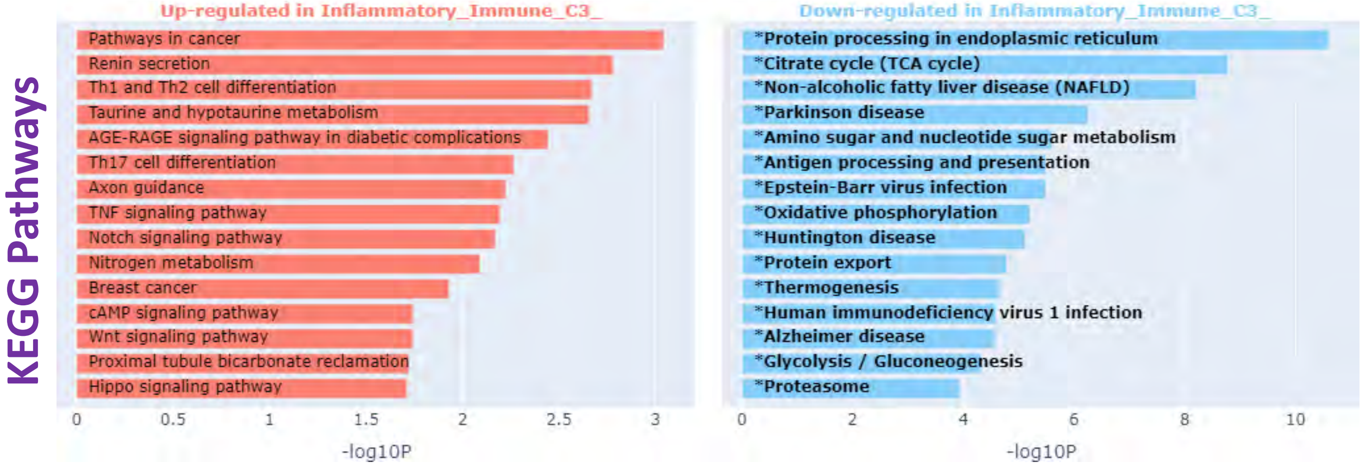

S3A.

CCA

B.

C.

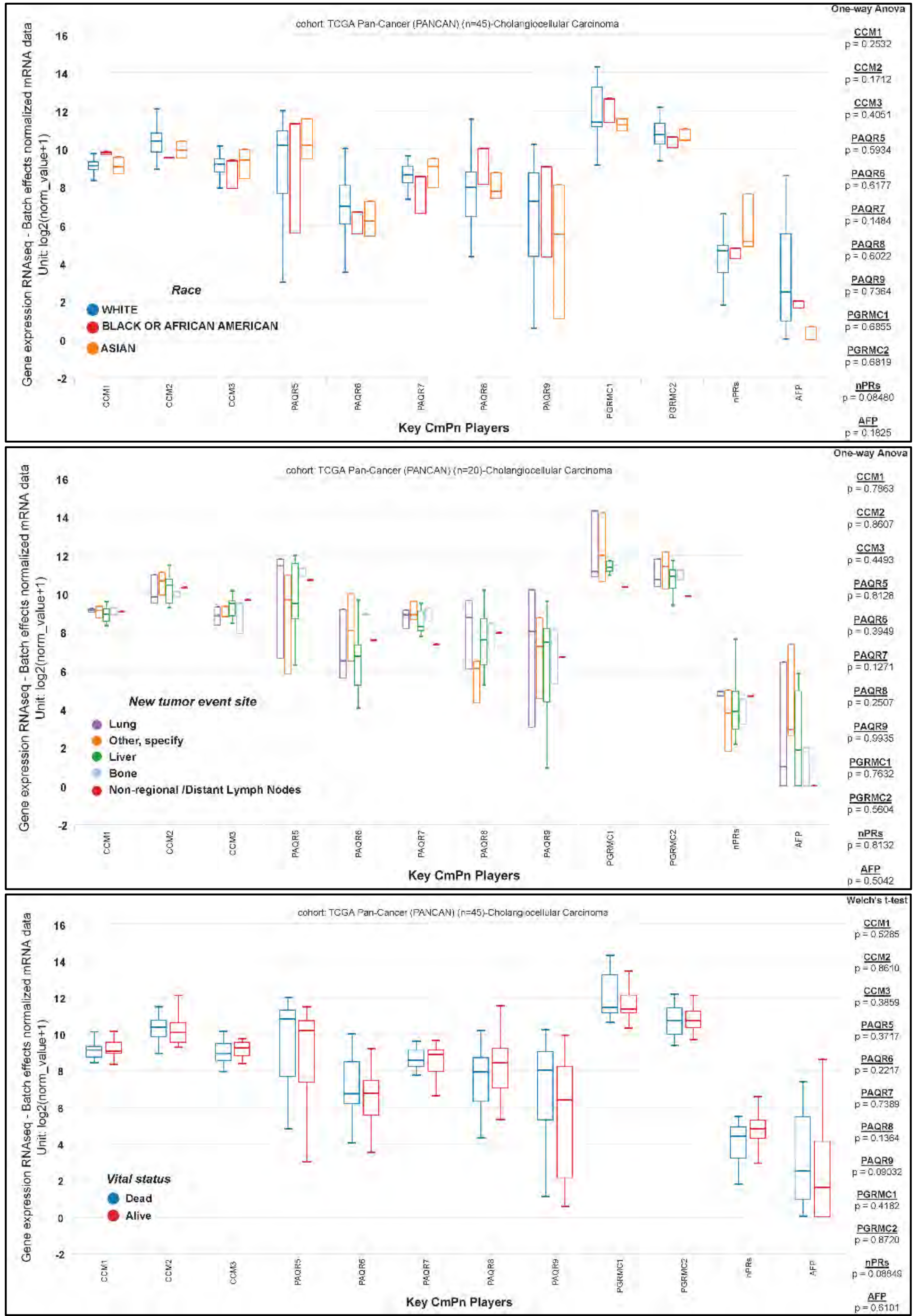
cohort: TCGA Pan-Cancer (PANCAN) (n=45)-Cholangiocellular Carcinoma
Gene expression RNAseq - Batch effects normalized mRNA data
Unit: log2(norm_value+1)
Race
WHITE
BLACK OR AFRICAN AMERICAN
ASIAN
CCM1
CCM2
CCM3
PAQR5
PAQR6
PAQR7
PAQR8
PAQR9
PGRMC1
PGRMC2
nPRs
AFP
Key CmPn Players
One-way Anova
CCM1 p = 0.2532
CCM2 p = 0.1712
CCM3 p = 0.4051
PAQR5 p = 0.5934
PAQR6 p = 0.6177
PAQR7 p = 0.1484
PAQR8 p = 0.6022
PAQR9 p = 0.7364
PGRMC1 p = 0.6855
PGRMC2 p = 0.6819
nPRs p = 0.08480
AFP p = 0.1825
cohort: TCGA Pan-Cancer (PANCAN) (n=20)-Cholangiocellular Carcinoma
New tumor event site
Lung
Other, specify
Liver
Bone
Non-regional /Distant Lymph Nodes
One-way Anova
CCM1 p = 0.7863
CCM2 p = 0.8607
CCM3 p = 0.4493
PAQR5 p = 0.8128
PAQR6 p = 0.3949
PAQR7 p = 0.1271
PAQR8 p = 0.2507
PAQR9 p = 0.9935
PGRMC1 p = 0.7632
PGRMC2 p = 0.5604
nPRs p = 0.8132
AFP p = 0.5042
cohort: TCGA Pan-Cancer (PANCAN) (n=45)-Cholangiocellular Carcinoma
Vital status
Dead
Alive
Welch's t-test
CCM1 p = 0.5285
CCM2 p = 0.8610
CCM3 p = 0.3859
PAQR5 p = 0.3717
PAQR6 p = 0.2217
PAQR7 p = 0.7389
PAQR8 p = 0.1364
PAQR9 p = 0.09032
PGRMC1 p = 0.4182
PGRMC2 p = 0.8720
nPRs p = 0.08849
AFP p = 0.6101

D-1. HCC

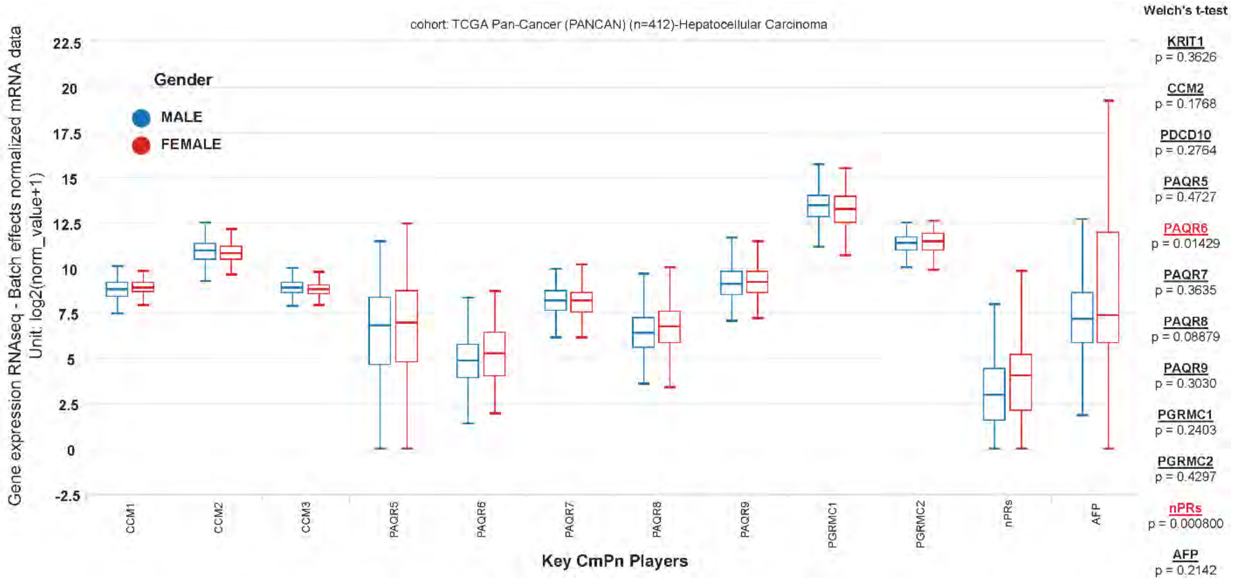

D-2. CCA

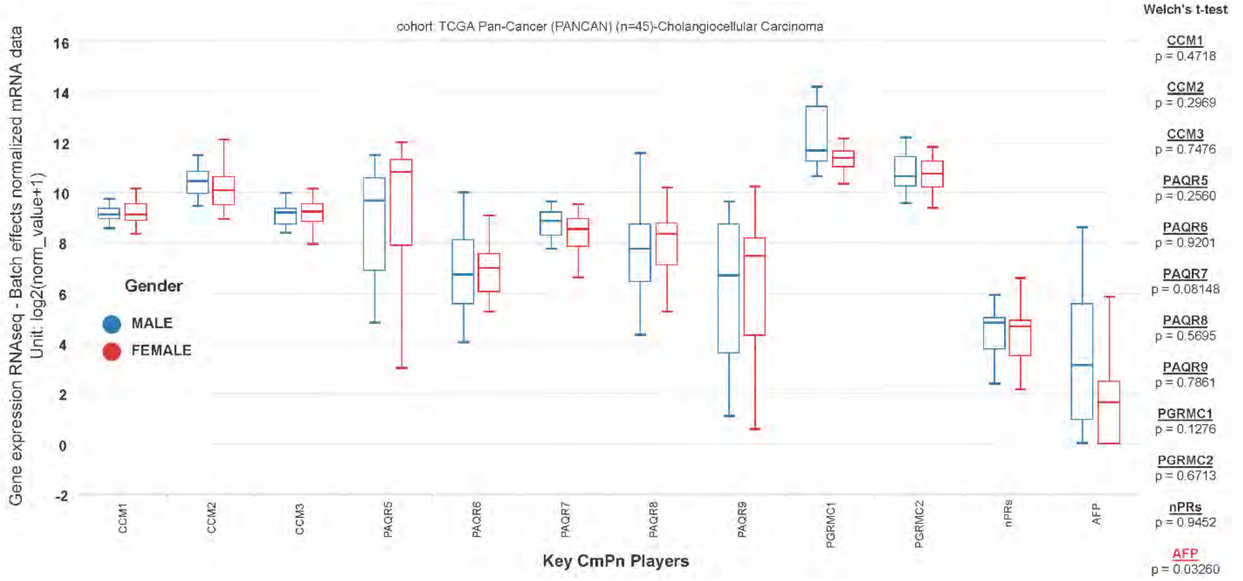

E-1.

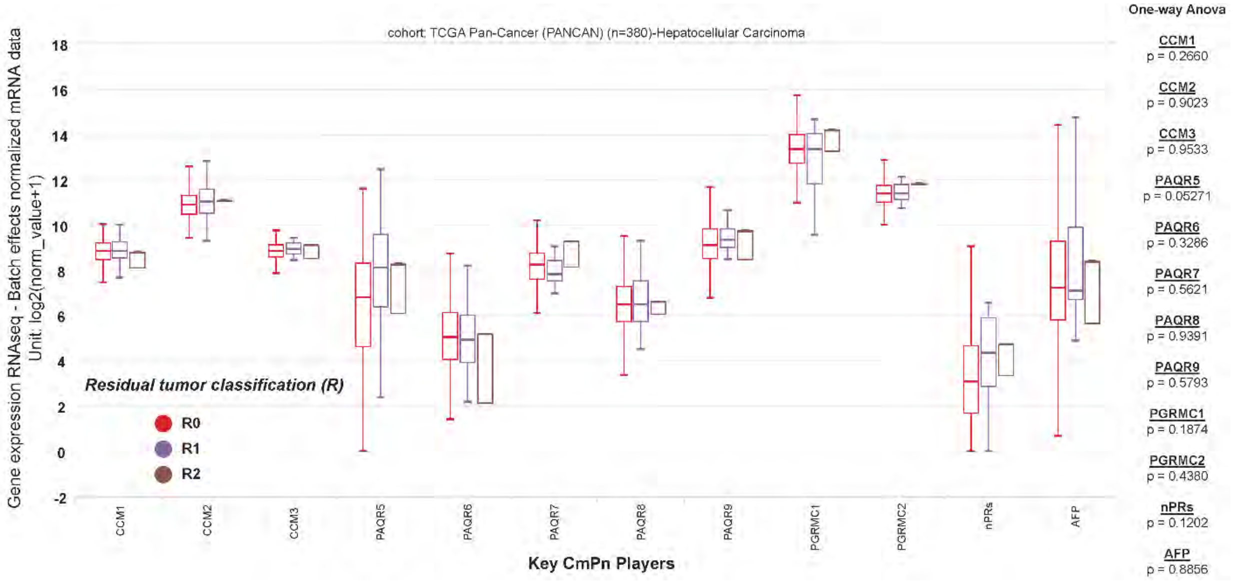

E-2.

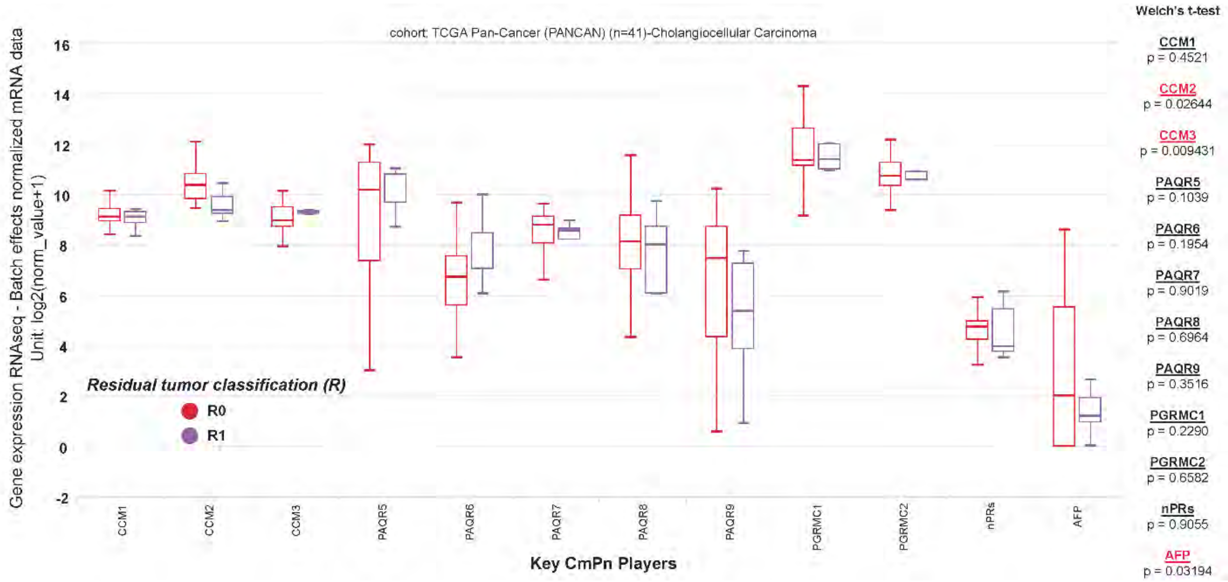

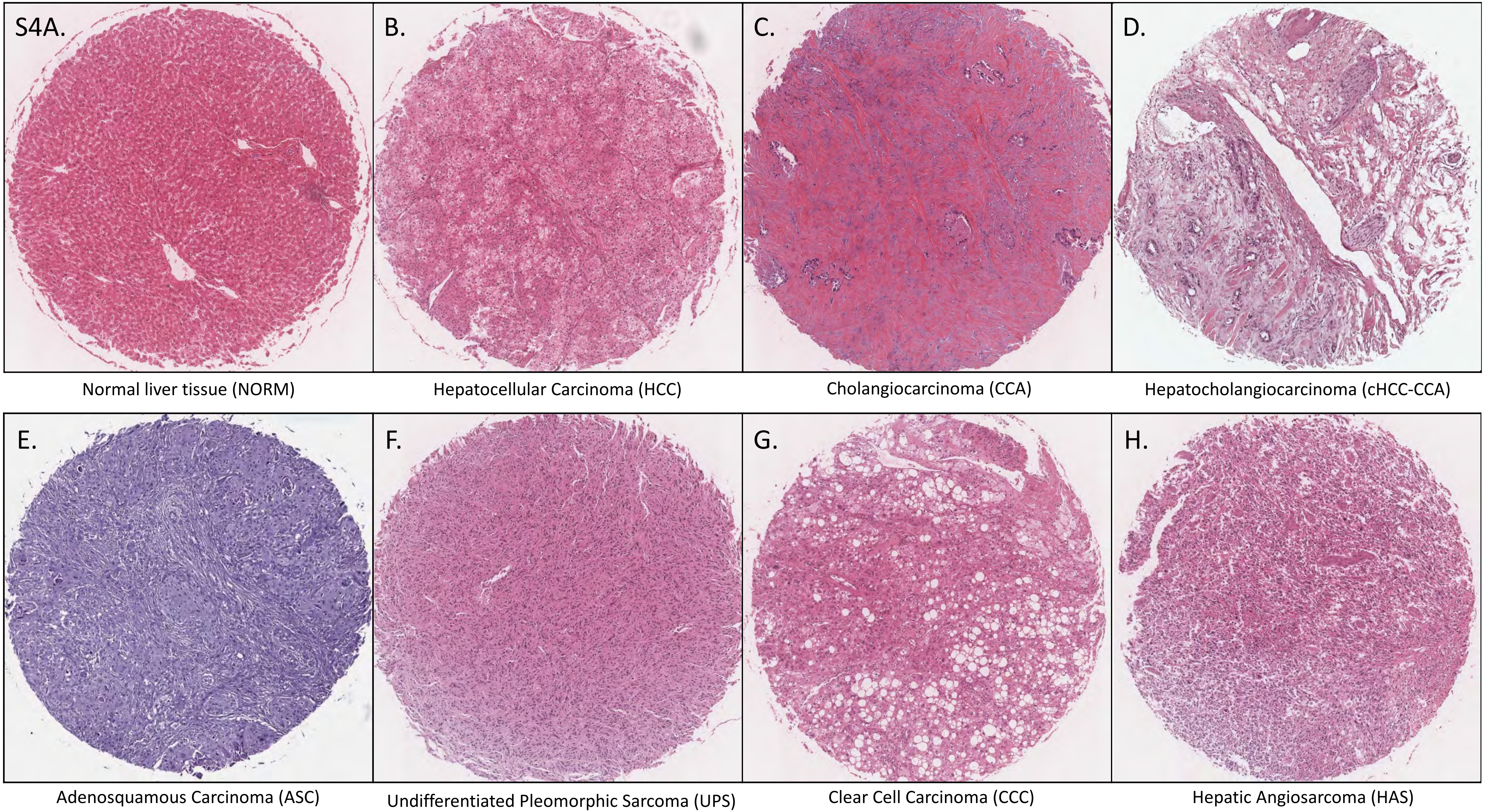
S4A.
B.
C.
D.
Normal liver tissue (NORM)
Hepatocellular Carcinoma (HCC)
Cholangiocarcinoma (CCA)
Hepatocholangiocarcinoma (cHCC-CCA)
E.
F.
G.
H.
Adenosquamous Carcinoma (ASC)
Undifferentiated Pleomorphic Sarcoma (UPS)
Clear Cell Carcinoma (CCC)
Hepatic Angiosarcoma (HAS)

S5A.

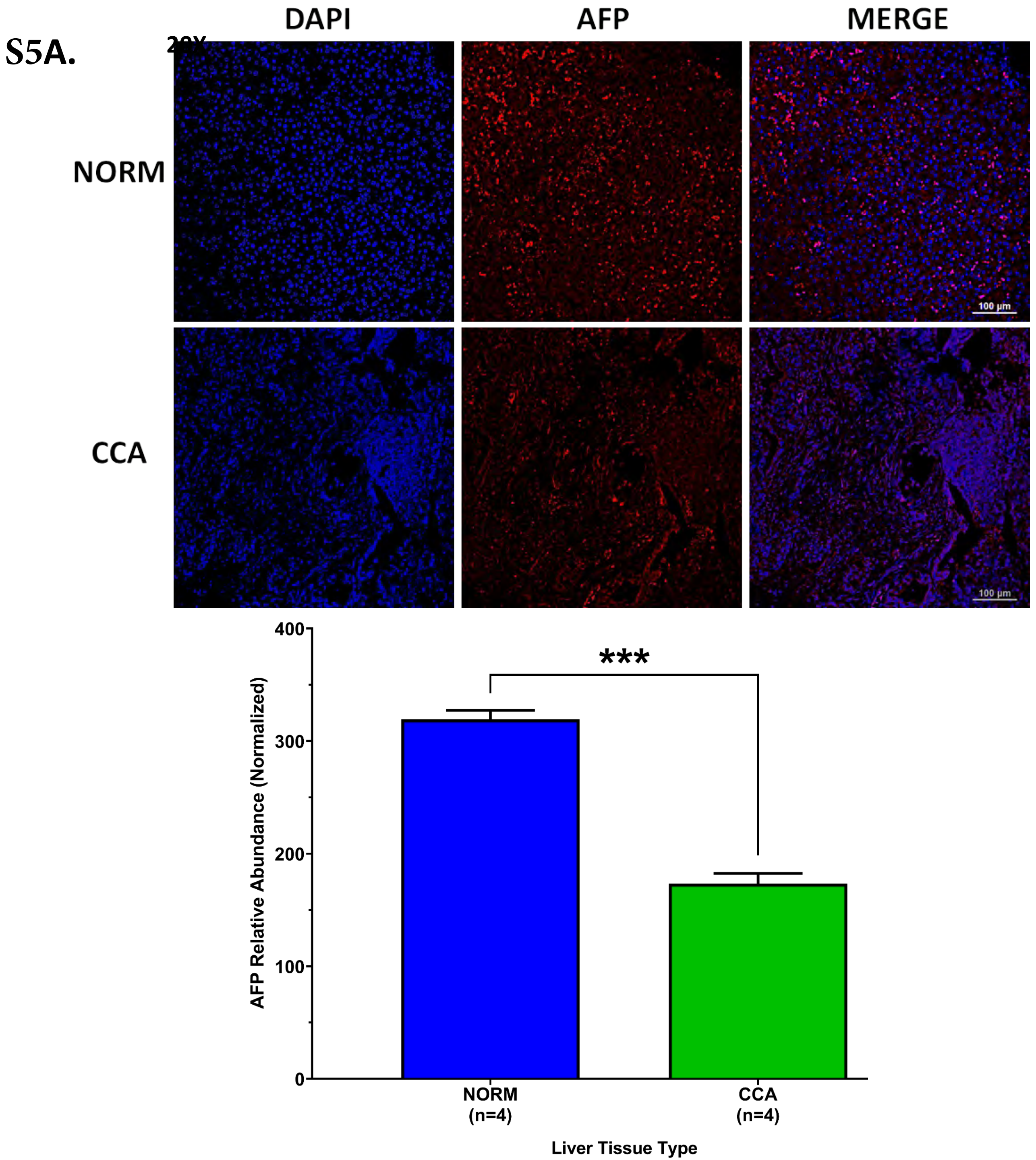

B.

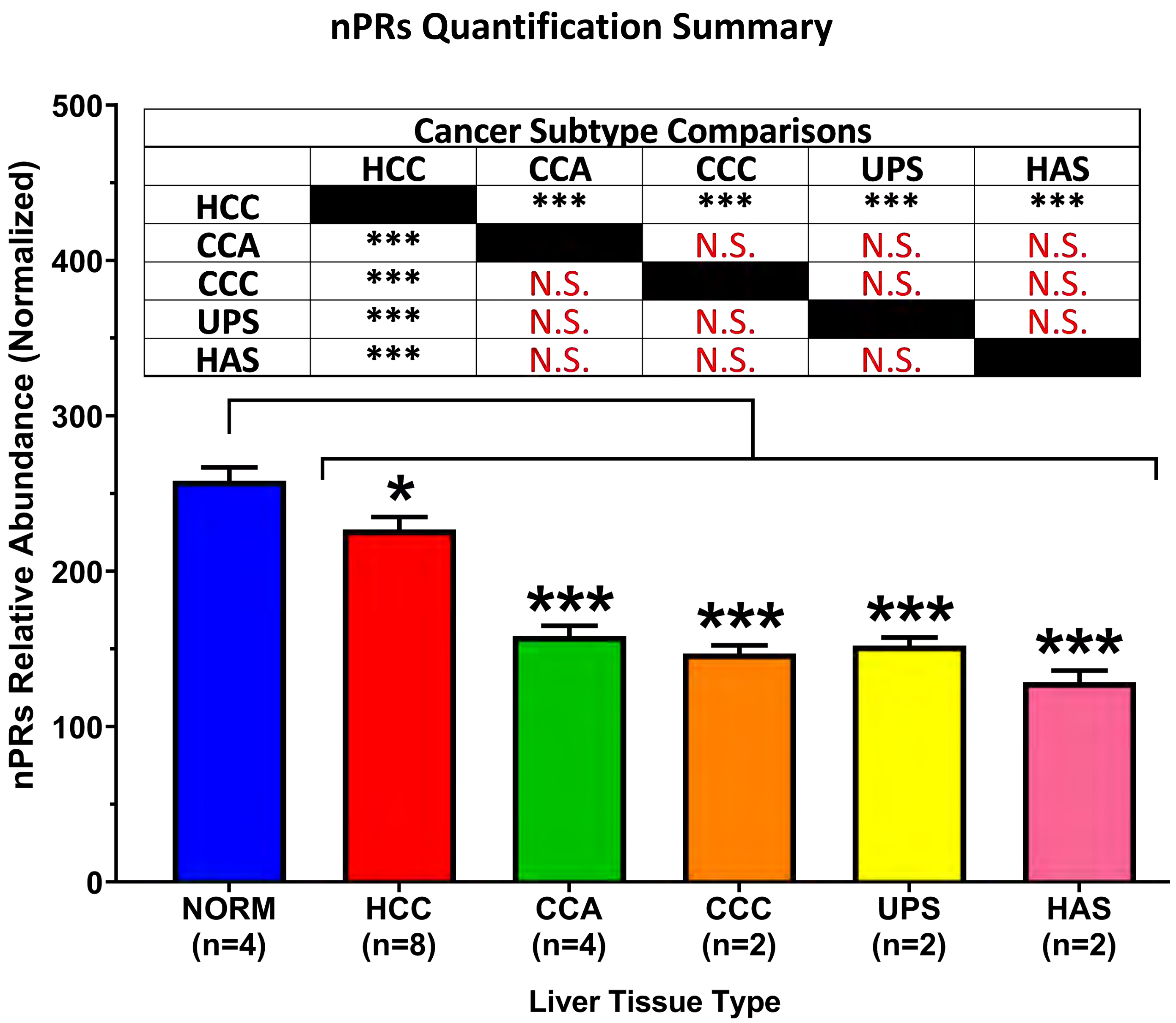

Cancer Subtype Comparisons					
	HCC	CCA	CCC	UPS	HAS
HCC		***	***	***	***
CCA	***		N.S.	N.S.	N.S.
CCC	***	N.S.		N.S.	N.S.
UPS	***	N.S.	N.S.		N.S.
HAS	***	N.S.	N.S.	N.S.	
